# A transparent and generalizable deep-learning framework for genomic ancestry prediction

**DOI:** 10.1016/j.ajhg.2026.06.003

**Published:** 2026-07-02

**Authors:** Camille Rochefort-Boulanger, Matthew Scicluna, Raphaël Poujol, Jean-Christophe Grenier, Pierre Luc Carrier, Sébastien Lemieux, Julie G. Hussin

**Affiliations:** 1 Research Centre, Montreal Heart Institute, Montreal, QC, Canada; 2 Département de Biochimie et Médecine Moléculaire, Université de Montréal, Montreal, QC, Canada; 3 Mila - Quebec Artificial Intelligence Institute, Montreal, QC, Canada; 4 Institute for Research in Immunology and Cancer, Université de Montréal, Montreal, QC, Canada; 5 Département de Médecine, Université de Montréal, Montreal, QC, Canada

**Keywords:** deep learning, genetic ancestry, population labels, biobanks, generalizability, interpretability, Diet Network

## Abstract

Accurately characterizing genetic ancestry is critical for ensuring reproducibility and fairness in genomic studies and downstream health research. This study aims to address the prediction of ancestry from genetic data using deep learning, with a focus on generalizability across datasets with diverse populations and on explainability to improve model transparency. We adapt the Diet Network, a deep-learning architecture proven to be effective in handling high-dimensional data, to learn population ancestry from single-nucleotide polymorphism (SNP) data using the populational Thousand Genomes Project dataset. Our results highlight the model’s ability to generalize to diverse populations in the CARTaGENE, Montreal Heart Institute, and *All of Us* biobanks and that predictions remain robust to high levels of missing SNPs. We show that, despite the lack of North African populations in the training dataset, the model learns latent representations that reflect meaningful population structure for North African individuals in the biobanks. To improve model transparency, we apply Saliency Maps, DeepLift, GradientShap, and Integrated Gradients attribution techniques and evaluate their performance in identifying SNPs leveraged by the model. Using DeepLift, we show that the model’s predictions are driven by population-specific signals consistent with those identified by traditional population-genetics metrics. This work presents a generalizable and interpretable deep-learning framework for genetic-ancestry inference in large-scale biobanks with genetic data. By enabling more widespread genomic ancestry characterization in these cohorts, this study contributes practical tools for integrating genetic data into downstream biomedical applications, supporting more inclusive and equitable healthcare solutions.

## Graphical abstract

**Figure:**
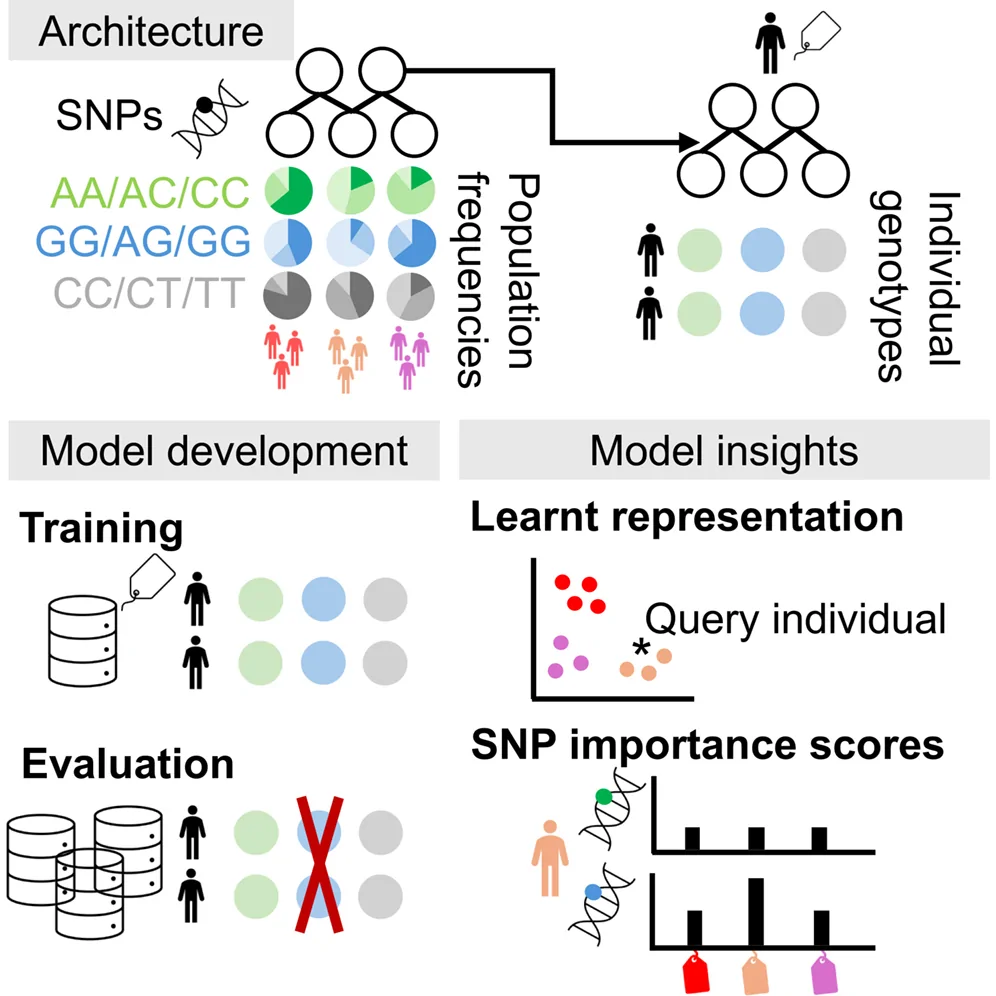

## Teaser

Using Diet Network models and explainable AI methods, we develop a robust framework for genetic-ancestry inference from SNP data across large-scale biobanks. The models generalize across datasets with differing SNP coverage and capture latent population structure, and explainable AI methods identify ancestry-informative genetic variants driving predictions.

## Introduction

With the rapid advancement of high-throughput sequencing and genotyping technologies,^1^^,^^2^ large-scale clinical cohorts and biobank datasets containing genetic data have become increasingly available, opening new opportunities for large-scale, data-driven health research.^3^ These resources have underscored the importance of including individuals from diverse genetic-ancestry groups to ensure the validity, reproducibility, and clinical relevance of genetic findings.^4^^,^^5^ In response, multiple initiatives have emerged to build biobanks that better represent populations historically underrepresented in genomic research.^6^^,^^7^^,^^8^ These efforts have also spurred interest in accurately characterizing population structure and inferring genetic ancestry, especially since population labels in biobanks are often based on self-reported ethnicity.^9^ Since such labels can be incomplete, imprecise, or constrained by predefined categories in questionnaires, genetic-ancestry inference from genomic data offers a complementary, biologically meaningful, and often more consistent alternative.^10^

Population labels have long been used in genetics to define reference populations, providing a framework for comparing genetic diversity across groups.^11^^,^^12^^,^^13^ These labels guide the study of population structure,^14^^,^^15^ evolutionary history,^16^^,^^17^ and disease susceptibility.^18^^,^^19^^,^^20^ Publicly available population reference datasets, such as HapMap,^21^ the Thousand Genomes Project (1KGP),^22^ and the Human Genome Diversity Project (HGDP),^23^ play a central role in human genetics by offering well-curated genetic data from individuals of diverse geographic and ancestral backgrounds. In these reference datasets, population labels are typically defined using a combination of geographic origin, linguistic background, and self-reported ancestry.

These population datasets have enabled the development of genetic-ancestry inference methods that learn population structure from reference panels and leverage these learned patterns to infer ancestry in other genomic datasets. Due to the high dimensionality of genomic data, many existing approaches first apply a dimensionality-reduction technique, such as principal-component analysis (PCA),^24^ followed by a clustering method or machine-learning classifier to predict genetic ancestry from a reduced set of features.^25^^,^^26^^,^^27^ In parallel, local-ancestry inference methods such as RFMix^28^ analyze genome-wide SNP data by training machine-learning models on phased genotype segments from a reference panel and subsequently inferring the ancestry of corresponding segments in target datasets. More recently, the rise of data-hungry deep-learning methods applied to such data has further expanded the methodological landscape of human genomics,^29^^,^^30^ including approaches for genetic-ancestry inference such as Orchestra,^31^ which also operates on segments of phased data but leverages more powerful deep-learning architectures.

While these methods are valuable tools for population-genetics experts, their broader adoption across the genetic research community remains challenging, despite the growing importance of accurate genetic-ancestry characterization. This limitation arises primarily from their limited generalizability across datasets, as these approaches rely on fixed sets of SNPs that will inevitably differ between cohorts. Applying these methods to new datasets requires access to curated reference panels to reselect SNPs followed by model retraining, which can be computationally intensive and demands technical expertise.

A further limitation of deep-learning models is their tendency to overfit when applied to genomic data, due to the “fat-data problem,” where the number of SNPs far exceeds the number of samples. While recent developments in deep learning, including transformer-based models and large language models, have demonstrated promise in diverse genomics applications,^32^^,^^33^^,^^34^ these models are designed for sequence data, capturing dependencies between nucleotides within an ordered genomic sequence and are not suited for genotyping array data. In contrast, the Diet Network is a deep-learning architecture specifically designed to handle high-dimensional genetic data while mitigating overfitting by using per-SNP summary statistics to constrain the network weights.^35^ Although prior studies have shown that the method can successfully infer genetic ancestry in the 1KGP and HGDP reference datasets,^35^^,^^36^ suggesting the feasibility of a ready-to-use trained model, its behavior on non-population genomic datasets and its robustness to variations in SNP coverage have not yet been characterized.

Another challenge in genetic-ancestry inference is the reliance on discrete population labels. While these labels enable group-level comparisons, they fail to capture the continuum of human genetic variation, particularly for individuals from admixed or underrepresented populations. This oversimplification may introduce biases in downstream analyses, including health-related predictions, and carries the risk of reinforcing discriminatory interpretations. In this context, interpretability becomes especially important; however, despite careful architectural design, deep-learning models are often criticized for their “black-box” nature, providing limited transparency into the specific SNPs driving their predictions.^37^

In this study, we address key challenges in genetic-ancestry inference by adapting the Diet Network to provide a framework that is generalizable both across datasets with varying SNP coverage and across diverse populations. We evaluated its generalizability on complete SNP data across a diverse spectrum of populations included in two biobanks, the Montreal Heart Institute Biobank (MHIB)^38^^,^^39^ and the CARTaGENE (CaG) biobank,^40^^,^^41^ which reflect the genetic heterogeneity of clinical cohorts. We demonstrated that the models maintain accurate predictions when applied to reduced sets of SNPs from downsampling simulations and application to real datasets with differing SNP coverage, such as the *All of Us* (AoU) biobank,^42^ highlighting their robustness to differences in genetic marker coverage across datasets. Additionally, we introduce a visualization method that captures how the model represents genetic diversity, an approach that uses the structure learned from the data to move beyond rigid population labels, extending the approach to individuals from admixed populations not represented in the training dataset. To improve transparency, we apply local feature-attribution methods to identify SNPs that are most predictive of population labels. By comparing these attributions with traditional population-genetics metrics, we gain a deeper understanding of how deep-learning models capture population structure. This framework aims to address the technical challenges associated with both the use of genetic data with explainable deep learning and the issues with population labeling in current datasets, contributing to more transparent and generalizable predictions in genomics.

## Methods

### Data processing

We obtained ethics approval for this study from the Montreal Heart Institute Institutional Review Board (IRB).

#### 1KGP dataset

The 1KGP whole-genome sequencing 30× coverage on the GRCh38 dataset^43^ was downloaded from the International Genome Sample Resource data portal (https://www.internationalgenome.org). The dataset includes whole-genome sequencing at 30× coverage of 2,504 samples categorized into 26 populations across five continents (Europe, East Asia, South Asia, Africa, and America). Table S1 lists the population descriptions and their corresponding three-letter abbreviation code.

We merged the population labels of closely related populations with minimal genetic differentiation: CEU and GBR (merged as CEUGBR), and ITU and STU (merged as STUITU), reducing the number of 1KGP populations from 26 to 24 (Notes S1.1). To mitigate class imbalance, CEUGBR and STUITU were downsampled randomly to match the largest population sample size within their respective continental groups (*n*_*CEUGBR*_ = 107; *n*_*STUITU*_ = 105).

SNP selection was performed as part of the construction of a harmonized SNP set designed to ensure complete overlap with CaG and MHIB, resulting in 229,986 SNPs (see construction of a harmonized SNP set in methods).

#### CaG datasets

The CaG platform comprises data collected using different genotyping arrays over time. The largest subset, which captures the greatest population diversity, includes 17,286 individuals genotyped with the Illumina Infinium Global Screening Array (GSA) v2 with the Multi-Disease Panel and the CaG_addon_v1_20037253_A2 array,^40^ hereafter referred to as the GSA subset. The platform also includes two earlier datasets: 990 individuals genotyped with the Affymetrix Axiom 2.0 UK Biobank array (Axiom_UKB_WCSG96) and 937 individuals genotyped with the Illumina HumanOmni2.5-8v1 Multi_A array, hereafter referred to as the Axiom and Omni subsets, respectively. These two subsets consist predominantly of French Canadian individuals. We accessed data through projects 771235 and 406713. Researchers can request access to CaG data by submitting a project proposal through the study website (www.cartagene.qc.ca).

Population labels for French Canadian individuals were assigned based on self-reported information: individuals had to identify as French Canadian, be of White European descent, and have all four grandparents born in Canada. This resulted in 9,437 French Canadian individuals genotyped on the GSA array, 772 on the Axiom array and 643 on the Omni array. For Italian, Chinese, Haitian, and Moroccan ancestry groups, individuals were included if they reported all four grandparents born in Italy (367 samples), China (107 samples), Haiti (230 samples), and Morocco (160 samples). These ancestry groups were restricted to individuals genotyped on the GSA array, as population diversity within the earlier genotyping platforms was limited. Additional details on population labels are provided in Methods S4.2.

For the GSA subset, SNPs selection was performed to ensure complete overlap with the 1KGP SNP set, resulting in 229,986 SNPs (see construction of a harmonized SNP set in methods). For the Axiom and Omni subsets, SNPs were selected based on their overlap with the harmonized SNP set shared between 1KGP and the CaG GSA subset, resulting in 170,538 SNPs for the Omni subset and 55,250 SNPs for the Axiom subset (see Methods S4.1).

#### MHIB dataset

We used data of 16,707 individuals genotyped on the GSA v3.0-MD genotyping array, the same version of the Illumina Infinium GSA array as the CaG GSA subset, as approved under project 2026-3546. Access to MHIB data is granted through a formal project submission process in collaboration with a researcher affiliated with the Montreal Heart Institute.

Population labels were primarily assigned based on the information available in the self-reported ethnicity field as grand-parental information was less consistently available compared to CaG. These initial assignments were further refined through genetic analyses: PCA was used to identify a subgroup of 105 individuals with North African ancestry among those who had self-identified as White, and individuals whose genetic ancestry estimated using RFMix v2^28^ did not align with their self-reported ethnicity were excluded (Methods S4.2).

For the French Canadian population, 12,884 individuals were included who self-identified as White and reported that both parents were of French Canadian descent. The African ancestry group included 117 individuals who reported having ancestors originating from Africa or Black ethnicity. The Asian ancestry group included 62 individuals who self-identified as Asian. Additionally, we identified a subset of 419 individuals of Italian descent, based on consistent grand-parental information indicating that all four grandparents were born in Italy.

SNP selection was performed to ensure complete overlap with the SNP set shared between 1KGP and the CaG GSA subset, resulting in 229,986 SNPs (see construction of a harmonized SNP set in methods).

#### AoU dataset

We used data of 447,278 individuals genotyped with the Illumina Infinium Global Diversity Array from the AoU Research Program’s Controlled Tier Dataset v8 available through authorized access on the Researcher Workbench. Population labels were assigned based on self-reported information in the race and ethnicity features defined by the biobank (Methods S4.2). In accordance with AoU data-sharing policies, we report only prediction profiles observed in at least 20 individuals. Individuals were classified as White (233,879 individuals; 231,168 reported), Black or African American (76,468 individuals; 74,511 reported), or Asian (14,230 individuals; 12,640 reported) if they reported the corresponding category in the race field of the questionnaire and did not report Hispanic or Latino in the ethnicity field. Individuals were classified as Hispanic or Latino (72,253 individuals; 69,284 reported in the paper) if they reported Hispanic or Latino in the ethnicity field and provided no information in the race field of the questionnaire.

SNPs were selected based on their overlap with the harmonized SNP set shared between 1KGP, the CaG GSA subset, and MHIB, resulting in 204,667 SNPs.

#### Construction of a harmonized SNP set

The SNP set used for model training was selected as the intersection across the 1KGP training dataset, the CaG GSA subset, and the MHIB dataset, providing a consistent input feature set that enable controlled comparison between complete and simulated incomplete SNP data. This set was obtained by first identifying the largest common set of SNPs shared between the CaG GSA and MHIB genotyping arrays. To build this maximal SNP set, the following steps were independently applied to the CaG GSA and MHIB datasets. Alleles were harmonized to the GRCh38 reference genome using Will Rayner’s strand alignment tool and the strand file GSAMD-24v3-0-EA_20034606_A1-b38 from https://www.strand.org.uk (last accessed on November 24, 2022). SNPs with allele frequency differences exceeding 20% compared to the TOPMed reference panel^44^ were removed, as they likely result from incorrect genomic conversion. SNPs with a missing rate above 10% were excluded and monomorphic sites were removed. The final maximal SNP set consisted of 566,791 SNPs, representing the intersection of the filtered SNPs from both CaG GSA and MHIB datasets.

The maximal SNP set shared between CaG GSA and MHIB was further intersected with the 1KGP dataset and additional filtering was performed using PLINK v1.9. SNPs with more than 10% missing rate or a minor allele frequency below 5% in 1KGP were removed. Linkage disequilibrium pruning was applied using the parameters –indep-pairwise 50 5 0.5. SNPs located in the human leukocyte antigen region were excluded (chromosome 6, positions 28,510,121 to 33,480,577 on GRCh38). These filtering steps resulted in a final set of 229,986 SNPs. Missing genotypes were subsequently imputed using ShapeIT5^45^ in cohort-based mode, without relying on an external reference panel.

### Diet Network architecture and training

The Diet Network consists of two jointly trained neural networks: a main network that performs population classification from individual genotype data and an auxiliary network designed to reduce overfitting that generates SNP-specific weights for a high-dimensional input layer using a SNPs embedding, a vector representation of every SNPs (Figure S1). As the original implementation^35^ was based on the now-deprecated Theano library, we reimplemented the architecture in PyTorch (see data and code availability section).

We trained 15 Diet Network models on the 1KGP dataset (2,322 individuals from 24 populations) using 5-fold cross-validation, repeating the process three times. Each fold was split into training (60%), validation (20%), and test (20%) sets, with population labels balanced between the splits. Genotype input features were standardized by subtracting the mean and dividing by the standard deviation calculated on genotypes from the training set. For the auxiliary network, SNP embeddings were defined as genotype frequencies per population in the training set. Hyperparameter tuning was conducted on the validation set. Further details on model training are provided in Methods S4.3.

Model training and evaluation were conducted on the Compute Canada Database (CCDB) of the Digital Research Alliance of Canada, except for evaluations on the AoU biobank, which were performed on AoU servers. After preprocessing the input data, models were trained using 10 GB of memory, eight CPU cores and a GPU (NVidia A100SXM4, 40 GB memory). Training a single model for 2,329 epochs took approximately 22 min (about 0.56 s per epoch). For evaluation on the CaG Axiom dataset (990 individuals), we used the same resources, and the total execution time, including preprocessing the data into HDF5 format, was 20 min. As GPU resources are not universally available, we additionally evaluated the method on the CaG GSA subset, the largest cohort included in our study (17,286 individuals), using CPU only. Relying on the pre-generated HDF5 file (which does not require a GPU), inference of genetic ancestry for all individuals required less than 3 min.

### Benchmarking

Two alternative approaches were used to infer genetic ancestry: a method based on PCA followed by random forest classification, adapted from the approach implemented by the Genome Aggregation Database (gnomAD) and hereafter referred to as the PCA+RF method, and the Rye method.

The PCA+RF method implemented by gnomAD is described in the literature^26^^,^^46^ and an example of the inference workflow is provided in gnomAD QC GitHub repository (https://github.com/broadinstitute/gnomad_qc/tree/main/gnomad_qc). The Rye method is described in Conley et al.,^47^ and the source code is available on GitHub (https://github.com/healthdisparities/rye).

The PCA+RF and Rye methods were evaluated using the same 1KGP dataset (2,322 individuals and 229,986 SNPs) and the same 5-fold split used to train the Diet Network. For the PCA+RF approach, PCA was performed using Scikit-learn version 1.7.1^48^ on the training set and, following the gnomAD shared protocol, the first 16 principal components (to match the number of principal components used by gnomAD) were used to train a random forest model using Scikit-learn RandomForestClassifier. Individuals in the validation and test sets were projected onto this PCA. The validation set was used to optimize the random forest hyperparameters, specifically the number of trees (1,000) and the maximum tree depth (10), while all other parameters were kept at the default values. Final model performance was evaluated on the test set of each fold. For Rye, we cloned and followed the instructions in the paper GitHub repository.^47^ The training set was used as the reference panel, and the validation set was used to tune the number of threads (final: 4) and iterations (final: 100). The final results were reported on the test set of each fold.

### Generalizability analyses

To assess the generalizability of the Diet Network, we performed both in-sample (1KGP) and out-of-sample (CaG GSA and MHIB) evaluations under varying levels of missing genotype data. In 1KGP, we simulated SNP missingness by removing predefined proportions of input features and compared classification accuracy across models trained with different input dropout rates (Methods S4.3).

Models trained with a 99.5% input dropout rate were applied to CaG (GSA, Omni and Axiom subsets), to MHIB and to AoU datasets, with prediction variability reported across 15 models per individual. Prediction shifts were tracked across varying proportions of missing SNPs compared to using complete SNP data in CaG GSA and MHIB datasets (Methods S4.3). In all experiments, the genotypes of the remaining SNPs were rescaled to account for the reduced input dimensionality resulting from SNP removal (Methods S4.4).

To examine how individuals are represented by the Diet Network models, we extracted activations from the last hidden layer (100 neurons) across all 15 trained models, resulting in a 1,500-dimensional representation per individual. These latent representations provide a compressed encoding of the input data learned by the network and can be extracted for any individual. To examine the learned population structure and potential out-of-distribution signals, we performed PCA (Scikit-learn 1.0.1) on the combined 1,500-dimensional latent vectors from all 1KGP individuals and projected the latent vectors of CaG GSA Moroccan and MHIB North African individuals onto this PCA space. The resulting principal components enabled joint visualization of how these groups relate to the training populations in the learned-representation space.

### Interpretability framework and baselines

We applied four feature-attribution methods using the Captum library: Saliency Maps, Integrated Gradients, DeepLift, and GradientSHAP. Each attribution method outputs local scores with dimensions SNP × population × individual, quantifying the contribution of each SNP to each predicted population class. For attribution methods requiring a baseline input (all except Saliency Maps), we designed several biologically informed single-sample and multi-sample baselines. All baselines matched the input dimensionality used to train the Diet Network models (229,986 SNPs). Single-sample baselines consisted of a single vector of this length, defined as follows:

• Genotype average: each SNP was assigned the mean genotype value computed across the training set. After input standardization (Methods S4.4), this baseline corresponds to a zero vector, effectively representing a sample with all genotypes missing.
• Reference: each SNP was set to the homozygous reference allele as defined in the GRCh38 reference genome. For biallelic SNPs, this corresponds to a genotype of 0.
• Highest-entropy sample: the individual from the training and validation sets whose model prediction yielded the highest entropy (i.e., most uniform probability distribution across the 24 1KGP population classes) was selected.

Each multi-sample baseline set was composed of 96 vectors:

• Random samples set: 96 individuals randomly selected from the training and validation datasets.
• Uniform set: 96 synthetic samples where each SNP position was assigned genotype values 0, 1, or 2 with equal probability ($p=\frac{1}{3}$).
• Global set: 96 synthetic samples where genotypes were drawn based on the global genotype frequencies observed across all 1KGP individuals.
• Per-population set: 96 synthetic samples where genotypes were drawn according to population-specific genotype frequencies from the 1KGP dataset, with four samples per population.

For all multi-sample baselines, attribution scores were computed individually for each sample in the set and then averaged.^49^

For each attribution method and each baseline, this resulted in a total of 229,986 × 24 local attribution scores per individual. These scores reflect, for each individual, the contribution of a given SNP to the predicted probability of every 1KGP population. Scores were computed for individuals in the test set of each of the 15 Diet Network models.

### Global and pairwise attribution scores

We aggregated local attribution scores to provide a single importance value per SNP across individuals. To compute these global attribution scores, we first selected the local attribution score computed with respect to the class predicted by the model for each individual. We then averaged these selected scores across individuals to obtain one global attribution score for each SNP.

We also computed pairwise attribution scores between true and target populations, reflecting how SNPs contribute to misclassification across groups. To compute these pairwise scores, individuals were first grouped by their true population. For each target population, we averaged the local attribution scores across all SNPs, yielding a single value per true-target population pair.

### Benchmarking attributions using SNP corruption and population differentiation

To assess whether attribution methods effectively identify important SNPs, we progressively corrupted input genotypes of individuals in the test sets based on attribution scores and measured the resulting change in classification accuracy. For each individual, SNPs were ranked according to their local attribution scores relative to the model’s predicted class and were corrupted in descending, ascending, or random order of importance using several corruption strategies.

We investigated several strategies for corrupting SNPs using DeepLift attribution scores.

• Shuffle corruption: for each corrupted SNP, genotype values were randomly permuted across the test set. This preserves the genotype distribution at each SNP position while disrupting individual-specific genomic patterns.
• 1NN imputation: corrupted SNPs were replaced with the corresponding genotypes from the individual’s nearest neighbor, identified using Euclidean distance on non-corrupted SNPs from the training and validation sets.
• Genotype-average corruption: corrupted SNPs were replaced by the corresponding values in the genotype-average baseline.
• Reference corruption: corrupted genotypes were replaced by those from the reference baseline.
• Highest-entropy sample corruption: corrupted values were drawn from the highest-entropy sample baseline.

For the genotype-average and reference corruption strategies, uncorrupted SNP values were rescaled (Methods S4.4).

To quantify the performance of attribution methods, the area under the accuracy curves was computed. These curves reflect how model accuracy changes as an increasing proportion of SNPs are corrupted. This evaluation yields two area metrics: *A*_*ord*_, corresponding to corruption in descending order of importance (from highest to lowest local attribution score), and *A*_*rev*_, for corruption in ascending order (from lowest to highest local attribution score).

Pairwise *F*_*ST*_ values^50^^,^^51^ were computed between all pairs of the 24 populations for all 229,986 SNPs from the harmonized SNP set using VCFtools 0.1.16. Pairwise *F*_*ST*_ matrices were generated for top SNPs identified with global attribution scores and randomly selected SNPs to assess correspondence between genetic differentiation and model-derived importance.

## Results

We used genetic data from four distinct sources (1KGP, CaG, MHIB, and AoU), each offering complementary strengths in terms of diversity, resolution, and real-world applicability. Diet Network models were trained on the 1KGP reference dataset, which includes samples from 26 populations, grouped into superpopulations representing five continental ancestry groups (Table S1). Models’ generalizability on complete SNP overlap was evaluated in CaG (GSA subset) and MHIB, two major Canadian research resources based in the province of Quebec that primarily include participants of French Canadian ancestry, a population of European descent. In addition to this majority group, both biobanks also include individuals from diverse genetic backgrounds reflecting the broader demographic landscape of the province. Generalizability under incomplete SNP overlap resulting from differences in genotyping protocols was assessed in the AoU biobank, a longitudinal cohort study that enrolls participants across the United States with a recruitment strategy explicitly aimed to increase the representation of non-European populations that have historically been underrepresented in biomedical research, as well as on two previous releases of the CaG cohort genotyped on different arrays (Axiom and Omni subsets).

### Training generalizable Diet Network models on 1KGP

Training models to distinguish genetically similar populations with distinct labels can lead to overfitting, where models rely on subtle allele frequency differences rather than capturing robust population structure, thereby reducing model generalizability. To address this, we merged the European CEU and GBR populations and the South Asian ITU and STU populations into single labels, CEUGBR and ITUSTU, respectively, resulting in a total of 24 populations. These merges were based on low pairwise *F*_*ST*_ values and supported by population structure and relatedness analyses (Notes S1.1 and Figure S2). Diet Network models were trained on 1KGP to classify 2,322 individuals into one of 24 populations using 229,986 SNPs. Training was performed using 5-fold cross-validation and three repetitions were analyzed, resulting in 15 models.

To improve the robustness of the models to missing genotypes, whether due to technical artifacts or non-overlapping SNPs across datasets, we trained the models using input dropout.^52^ This regularization technique randomly masks input feature information (genotypes) during training to prevent the model from relying too heavily on a small subset of informative SNPs. Models were trained with varying input dropout rates, and their ability to handle missing SNPs was assessed by measuring classification accuracy on a held-out test set, in which missing values were simulated by randomly removing increasing proportions of SNPs (Methods S4.3).

**Figure 1:**
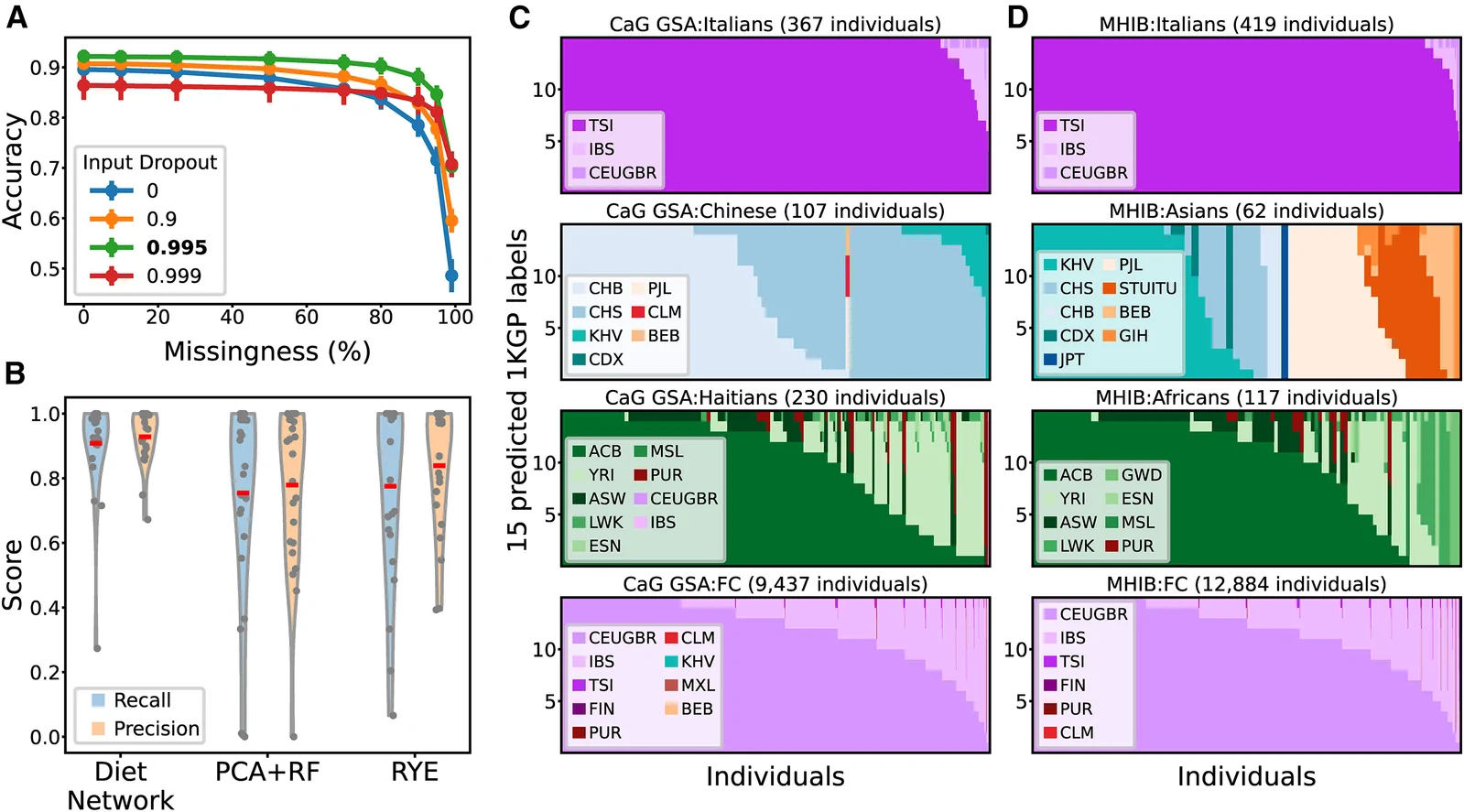
Diet Network generalization (A) Classification accuracy (*y* axis) of the Diet Network models under increasing proportions of missing SNPs (*x* axis), evaluated for input dropout rates 0, 0.9, 0.995, and 0.999. Accuracies at 0% missingness are averaged over 15 models, while accuracies for non-zero missingness proportions are averaged over the same 15 models and 100 distinct random SNP removal sets. Error bars represent the standard deviation across the 15 models. (B) Precision and recall scores per population for the Diet Network, PCA+RF, and Rye methods. Each point represents a population and the mean value is indicated by the red line. (C and D) Predicted population labels for CaG (C) and MHIB (D) individuals. Each vertical line represents an individual and the *y* axis shows their 15 population labels predicted by the 15 models. FC, French Canadian individuals.

Models trained with input dropout demonstrated improved performances and a greater robustness to missing values (Figure 1A). More precisely, an input dropout rate of 0.995, retaining an average of 1,150 SNPs per sample during training, yielded the best accuracy on the complete test set and maintained higher accuracy even with up to 99% simulated missing SNPs. Classification results by population are shown in a confusion matrix in Figure S3.

### Benchmarking Diet Network against existing methods

We compared the Diet Network with two alternative genetic-ancestry inference methods. To ensure a fair comparison, we excluded more complex local-ancestry inference approaches, such as RFMix and Orchestra, which require phasing a reference panel. Specifically, we benchmarked the Diet Network against a PCA+RF method derived from the one provided by gnomAD and against the Rye method, which has previously been applied to study populations in the AoU biobank. Both methods were evaluated on the 1KGP dataset using the same folds employed for training the Diet Network. The Diet Network achieved higher mean recall and precision across the 24 populations compared to PCA+RF and Rye (Figure 1B). The largest performance differences between the Diet Network and the two other methods were observed in African populations, where PCA+RF and Rye struggled to distinguish ESN from YRI, and in East Asian populations, where both methods confused China (CDX) and Vietnam (KHV) populations (Figure S4). The only exception was the African American ASW population, where the Diet Network performed worse than PCA+RF, misclassifying ASW samples as the African American ACB population.

### Validating generalizability in biobanks

We assessed the generalizability of Diet Network models trained on the 1KGP dataset by applying the best-performing models (trained with 99.5% input dropout) to individuals with diverse self-reported ancestries from the CaG, MHIB, and AoU datasets.

While 1KGP assigns populations using well-defined inclusion criteria, biobanks typically rely on self-reported ethnicity, which can vary in accuracy and resolution as it relates to genetic ancestry. To navigate this, we implemented strategies to assign population labels tailored to the available metadata in CaG, MHIB, and AoU, as described in methods and Methods S4.2.

#### Model predictions capture continental ancestry

Generalizability of the 15 Diet Network models was evaluated on complete SNP data from the CaG GSA subset and MHIB for Italian, Asian, African, and French Canadian populations. For individuals of Italian descent, model predictions in both CaG and MHIB, were consistently assigned to the corresponding Italian TSI population in 1KGP, with some models occasionally assigning nearby European populations (Figures 1C and 1D, Italians). Similarly, predictions for Chinese individuals in CaG aligned with 1KGP East Asian populations (Figure 1C, CaG GSA:Chinese). In MHIB, the broad self-reported Asian category encompassed both East and South Asian ancestries, reflected in the model predictions, which spanned East Asian and South Asian superpopulations while consistently agreeing at the continental level for each individual (Figure 1D, MHIB:Asian). Individuals of African or African-European admixed ancestry, such as Haitians in CaG and self-identified Africans in MHIB, were generally predicted as belonging to admixed African-Caribbean (ACB) or West African (YRI) populations, with occasional predictions of ASW, Puerto Rican (PUR), and other African ancestries (Figures 1C and 1D). The concordance of predictions across biobanks suggests similar representation of the underlying admixed ancestries for Haitian individuals, which was confirmed with grand-parental information (Notes S1.2) and demonstrates that the approach successfully captures an admixed ancestry not explicitly present in the training set.

The French Canadian founder population, the Western European majority group in both CaG and MHIB but absent from 1KGP, provided an opportunity to assess how the model generalizes to a well-represented but unmodeled population. French Canadian individuals were typically predicted as CEUGBR or IBS (Figures 1C and 1D), illustrating the model’s capacity to interpolate across unrepresented but related groups.

Overall, across all examined populations, model predictions largely aligned with their continental ancestry, underscoring the generalizability of the model, while also capturing finer-scale patterns and uncertainties reflective of real-world population structure and labeling.

#### Model predictions remain consistent on incomplete SNP data

**Figure 2:**
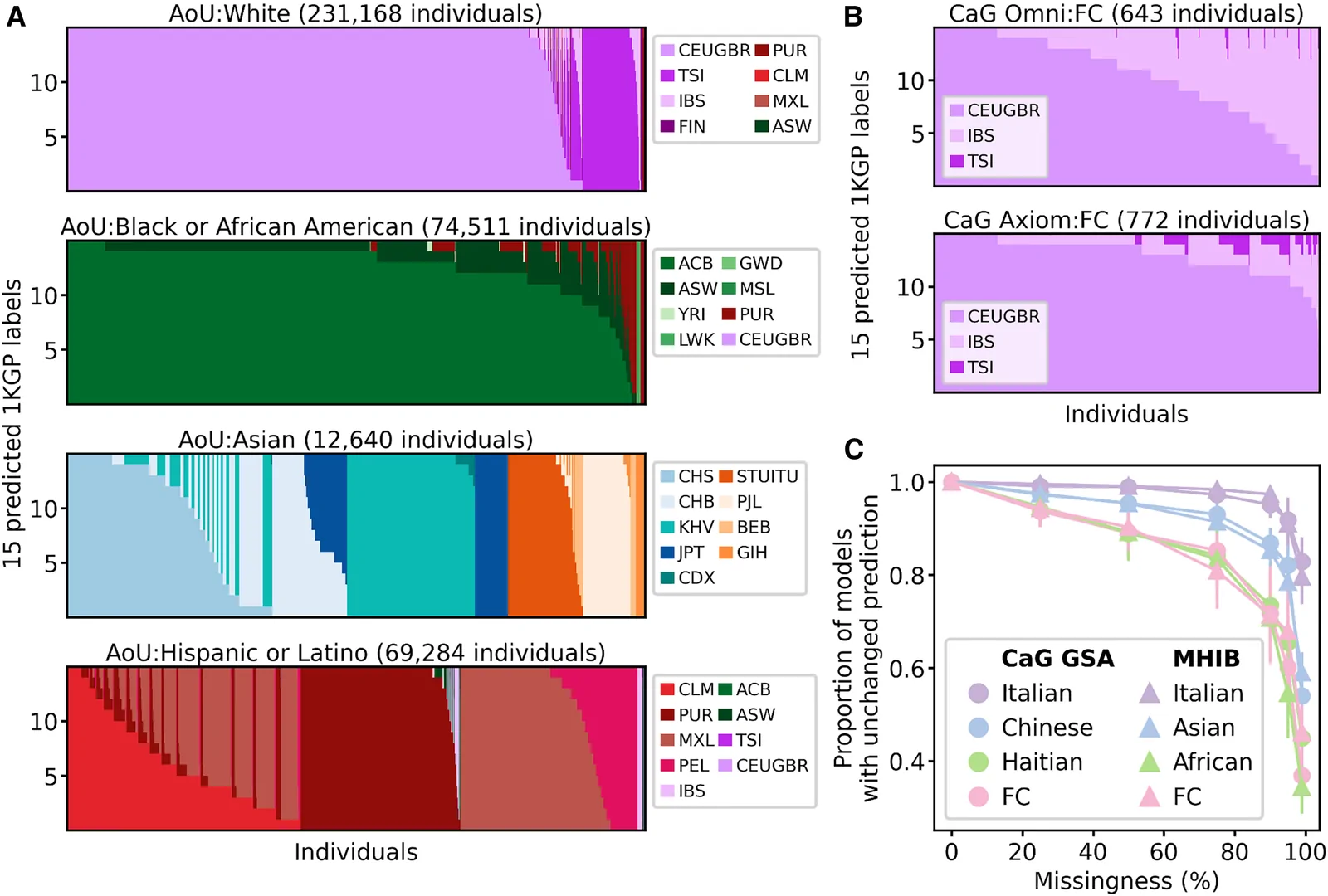
Diet Network generalization on incomplete data (A and B) Predicted population labels for AoU (A) and CaG Omni and Axiom (B) individuals. Each vertical line represents an individual and the *y* axis shows their 15 population labels predicted by the 15 models. FC, French Canadian individuals. (C) Proportion of predictions that match those obtained with complete SNP data (*y* axis), evaluated for increasing levels of SNP missingness (*x* axis) for each CaG GSA and MHIB population, considering all 15 predictions per individual. Error bars represent the standard deviation across individuals in each CaG GSA and MHIB population.

Generalizability of the 15 Diet Network models under incomplete SNP overlap was evaluated using the AoU cohort. Relative to the full training set, the dataset contained 25,319 missing SNPs, representing approximately 11% missingness. Despite this incomplete SNP overlap, model predictions largely captured continental ancestry. Individuals self-reporting as White were predominantly assigned to European populations, those reporting as Asian were consistently classified by the 15 models as East or South Asian, and individuals identifying as Black or African American were assigned to African-admixed and African populations, with a subset of models assigning individuals to the PUR population (Figure 2A). The AoU cohort additionally includes a population category not present in CaG or MHIB: individuals self-reporting as Hispanic or Latino. For this group, model predictions were largely assigned to 1KGP admixed populations from the Americas, including PUR, MXL, CLM, and PEL with a small number of models assigning European and African American populations (Figure 2A, Hispanic or Latino). Overall, Diet Network models generalize well to an external, more diverse cohort and maintain coherent ancestry assignments even when trained and evaluated on datasets with incomplete SNP overlap.

Generalizability of the 15 Diet Network models under higher levels of incomplete SNP overlap was assessed using the CaG Axiom and Omni subsets for the French Canadian population. Relative to the full training set, the Omni subset contained 59,448 missing SNPs (approximately 26%), whereas the Axiom subset contained 174,736 missing SNPs (approximately 76%). Despite these high levels of missing data, the prediction profiles for French Canadian individuals (Figure 2B) were similar to those obtained using complete SNP data in the CaG GSA subset (Figure 1C), indicating that model predictions for this population remain stable despite substantial SNP missingness.

To quantify the changes in model predictions under missing-data conditions, we incrementally introduced missing SNPs into the complete CaG GSA and MHIB datasets and compared the resulting predictions to those obtained using complete SNP data. Figure 2C shows, for each investigated population in CaG GSA and MHIB, the proportion of models whose predictions differed from those generated using complete data. Prediction changes varied by population: predictions for Italian individuals were the most consistent across the different levels of SNP missingness, while greater instability was observed among predictions for CaG Haitians, MHIB Africans, and French Canadian individuals in both cohorts. This suggests that model predictions are more susceptible to missing values for admixed populations and populations lacking a closely related reference in 1KGP. However, despite differences across populations, the change in predictions generally remained within the same continental population with notable shifts occurring only near 99% missingness (Figure S5). Furthermore, examining prediction variability across increasing levels of missing data highlights the improved generalizability achieved through input dropout training, as models incorporating input dropout produced more consistent predictions across increasing amounts of missing genotypes than those trained without input dropout (Figure S6).

#### Hidden representations provide insights into unseen populations

**Figure 3:**
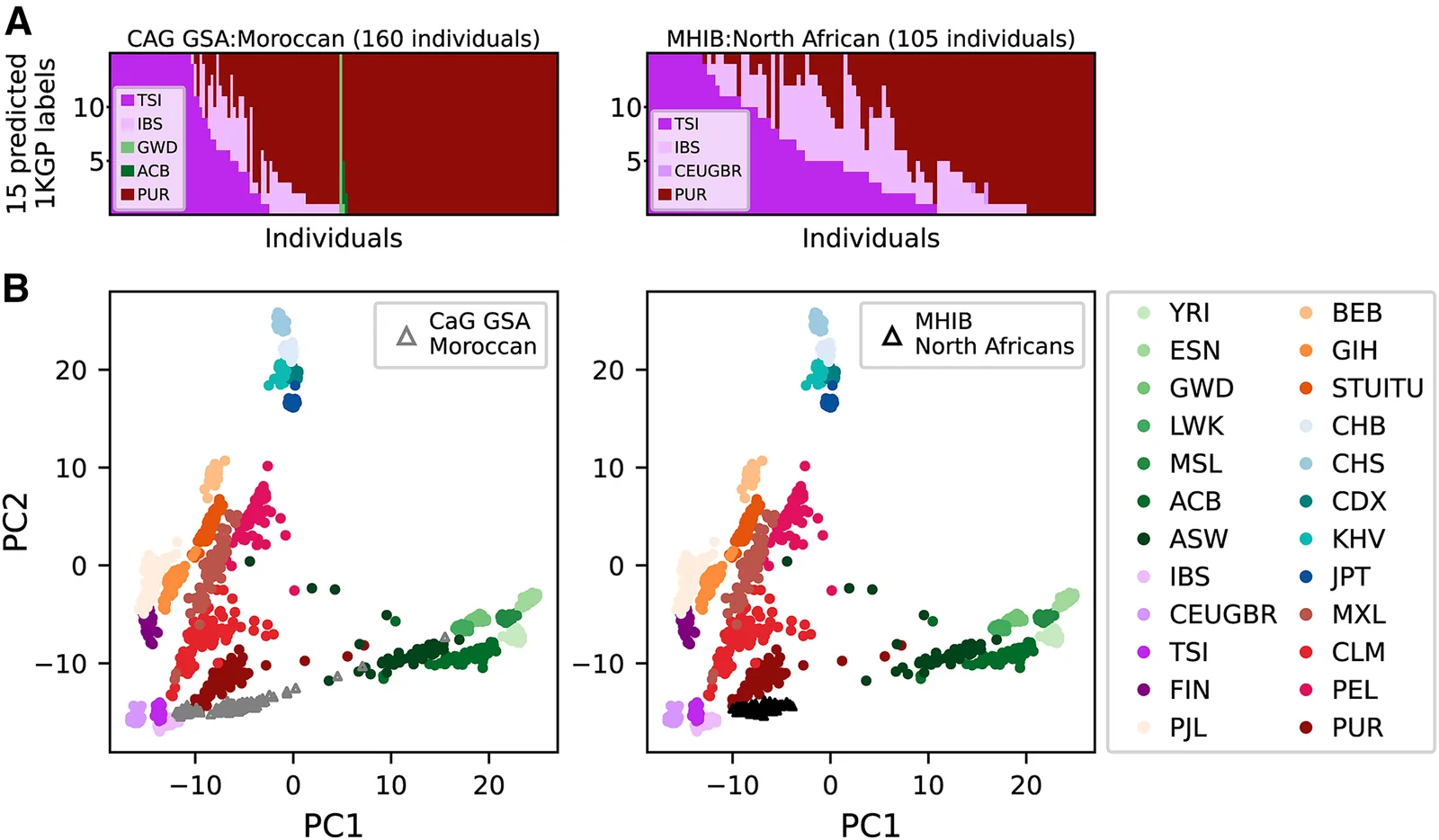
Diet Network hidden representation (A) Predicted populations for individuals of Moroccan ancestry in CaG GSA and North African ancestry in MHIB. Each vertical line represents an individual and the *y* axis shows the population predicted by each of the 15 models. (B) Final-layer PCA visualization for the 15 models based on 1KGP dataset with projected embeddings of Moroccan individuals in CaG GSA and of North African individuals in MHIB.

The 1KGP dataset does not include any North African reference populations, while both CaG and MHIB include participants who self-identify as North Africans, including a subset of individuals in CaG GSA reporting Moroccan ancestry. This presents a unique challenge: individuals from these populations constitute out-of-distribution samples, as they lack close genetic counterparts among the training labels, limiting the model’s ability to assign meaningful predictions. Nevertheless, by design, the model must assign each individual to one of the populations represented in the training set. For Moroccan individuals in CaG GSA and North African individuals in MHIB, predictions were distributed across PUR, TSI, and IBS (Figure 3A).

To better understand how the model handles such out-of-distribution samples, we examined the learned representations of these participants in the final hidden layer of the Diet Network models. We extracted the final hidden layer representations for 1KGP samples and performed PCA on these representations to construct a reference representation space. The same layer was extracted for CaG GSA and MHIB North African individuals, which were then projected onto this PCA space, to visualize how the model positioned them relative to the 1KGP populations. These samples do not overlap with any specific 1KGP population, indicating that the model captures a distinct genetic profile rather than collapsing these individuals into an existing reference group (Figure 3B). Instead, like PUR samples, they are positioned between individuals of European and African ancestries, consistent with their expected genetic ancestries.

### Uncovering model-leveraged SNPs using interpretability

Understanding which genetic variants the Diet Network models rely on can help us evaluate how it makes predictions. We applied feature-attribution gradient-based methods Saliency Maps,^53^ DeepLift,^54^ GradientSHAP,^55^ and Integrated Gradients^56^ to estimate the importance of individual SNPs on the model’s predictions.

#### Evaluating feature-attribution methods

DeepLift, GradientSHAP, and Integrated Gradients require a baseline input, which serves as a reference point to measure the effect of each SNP. Here, we use a genotype-average baseline, where each SNP is assigned the average genotype value across the training set. Because all inputs are standardized using *Z* score normalization before being passed to the network, this baseline input effectively becomes a vector of zeros, which can be interpreted as representing the absence of genetic information at each position.

The different gradient-based methods all produce local importance scores that we refer to as local attribution scores. To evaluate whether these local attribution scores identify the SNPs effectively used by the model, we examined how corrupting SNPs with high versus low attribution scores affects classification accuracy. Under the expectation that the attribution scores identify informative SNPs, corrupting SNPs with high attribution should lead to a more rapid decline in model accuracy than corrupting SNPs with low attribution.

For each individual, we ranked the SNPs using their local attribution scores computed with respect to their predicted population. We progressively corrupted increasing numbers of SNPs based on their ranking, either starting with the highest scoring SNPs (ordered corruption) or the lowest scoring SNPs (reverse corruption). SNP corruption was performed by shuffling genotypes across individuals, referred hereafter as “shuffle corruption.” This approach disrupts the informative signal available to the model while preserving the overall genotype distribution.

**Figure 4:**
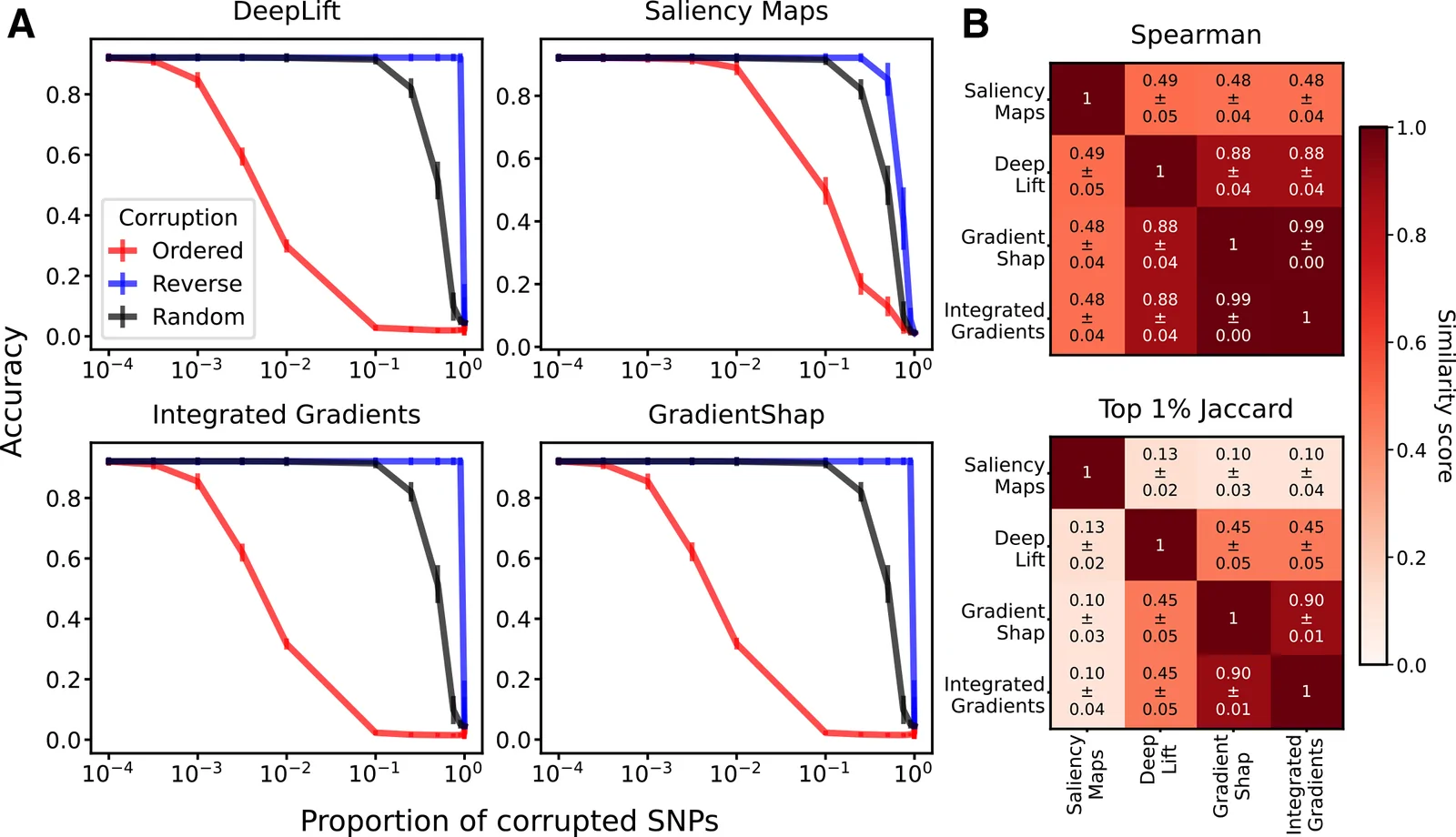
Performance of feature-attribution methods and baselines (A) Diet Network models classification accuracy on test sets for increasing proportions of corrupted features by feature-attribution method. Error bars indicate the standard deviation across samples and the 15 Diet Network models. (B) Spearman correlation and top 1% Jaccard index comparing the similarity of attributions computed using between feature-attribution methods (using the average-genotype baseline when applicable).

For all attribution methods, the ordered corruption led to a more rapid degradation of classification accuracy versus corrupting the same numbers of SNPs in the reverse order or at random (Figure 4A). The degradation of classification accuracy was quantified using the area under the accuracy curves (AUC) shown in Figure 4A. This resulted in two area metrics per method: the area under the ordered corruption curve (*A*_*ord*_), which should be small to indicate a rapid decline in accuracy, and the area under the reverse corruption curve (*A*_*rev*_), which is expected to be larger, reflecting a more gradual decrease in accuracy. Mean AUC values and standard deviations across the 15 models for each attribution method, along with the AUC obtained when removing SNPs at random (*A*_*ran*_), are reported in Table 1 and were used to evaluate the performance of the attribution methods. For each attribution method, *A*_*ord*_ was consistently lower than *A*_*ran*_ across all 15 models, with a mean fold decrease >12, while *A*_*rev*_ was consistently larger than *A*_*ran*_, with mean fold increase >1.8. Importantly, these effects were accompanied by low inter-model variability, as reflected by the small standard deviations across replicates. This indicates that local attribution methods identified SNPs that are important to the model’s prediction. In addition, the AUC values revealed that Saliency Maps underperformed the other attribution methods, with a >5.5-fold decrease in *A*_*ord*_ and >1.3-fold increase in *A*_*rev*_.

**Table 1:** Area metrics comparison of feature-attribution methods

**Method**	***A***_***ord***_	***A***_***rev***_
DeepLift	0.039 ± 0.008	0.877 ± 0.013
GradientShap	0.036 ± 0.006	0.878 ± 0.013
Integrated Gradients	0.036 ± 0.007	0.878 ± 0.013
Saliency Maps	0.201 ± 0.020	0.653 ± 0.035

AUCs obtained with ordered SNP corruption (*A*_*ord*_), reverse SNP corruption (*A*_*rev*_), and random SNP corruption (*A*_*ran*_ = 0.481 ± 0.025).

To assess the consistency between attribution methods, we computed the Spearman correlation on the local attribution scores. Additionally, we used the Jaccard index on the top 1% of high-attribution SNPs to evaluate the overlap of important SNPs identified by different methods. The three top-performing methods, DeepLift, GradientSHAP, and Integrated Gradients, largely agreed on the SNP rankings (Figure 4B). As no single method consistently outperformed the others, we selected DeepLift for subsequent analyses as a representative, well-performing approach.

#### Selecting an appropriate input baseline

We investigated how the choice of the input baseline influences the performance of the feature-attribution method. Prior studies suggest that an effective baseline should resemble other samples in the dataset, reflect an absence of information, and produce neutral predictions.^56^ With this in mind, we designed three single-sample baselines tailored to our genetic context (detailed in the methods section): (1) a genotype-average baseline, described above; (2) a reference baseline, homozygous for alleles in the human reference genome; and (3) a highest-entropy sample baseline, corresponding to the sample that yields the most uniform predictions across populations.

In addition to these single-sample baselines, we explored averaging contributions across multiple baselines, which can help reduce bias from any single point of comparison.^57^ These included a set of randomly selected real samples and three synthetic sets, where genotypes were generated according to different distributions: (1) a uniform frequency set, where genotypes are drawn with equal probability ($p=\frac{1}{3}$); (2) a global frequency set, where genotypes are sampled based on their frequency in the dataset; and (3) a per-population frequency set, where genotypes are sampled per population according to that population’s genotype frequencies.

We evaluated the baseline performance using the *A*_*ord*_ and *A*_*rev*_ metrics under two corruption methods: the shuffle corruption method described above and a 1 nearest neighbor (1NN) imputation corruption method where genotypes are replaced by the genotypes of the nearest neighbor in the dataset (see methods).

Among the single-sample baselines, the genotype-average baseline yielded the best performance, reflected by the lowest *A*_*ord*_ and highest *A*_*rev*_ values (Table 2). Among the multi-sample baseline sets, the randomly selected samples, global frequency, and per-population frequency baselines performed similarly, while the uniform frequency baseline performed worse. This likely stems from the fact that sampling genotypes uniformly, without adjusting for the actual genotype frequency distribution observed in the dataset, produces unrealistic inputs diverging from true individual genetic profiles. Furthermore, we observed that each single-sample baseline achieved the best performance when SNPs were corrupted by replacing their values with those of that same baseline, highlighting the importance of assessing baseline performance across multiple corruption methods (Table S2). We also observed lower *A*_*ord*_ and *A*_*rev*_ performance when using suboptimal baselines, confirming that the area metrics can identify suboptimal baselines (Figure S7).

**Table 2:** Area metrics comparison across baselines and corruption methods

	**Shuffle corruption**		**1NN imputation**	
Baseline	*A*_*ord*_	*A*_*rev*_	*A*_*ord*_	*A*_*rev*_
Reference	0.049 ± 0.008	0.871 ± 0.013	0.046 ± 0.006	0.753 ± 0.014
Genotype-average	0.039 ± 0.008	0.877 ± 0.013	0.039 ± 0.006	0.84 ± 0.014
Highest-entropy sample	0.048 ± 0.008	0.875 ± 0.013	0.045 ± 0.006	0.796 ± 0.016
Set: random samples	0.04 ± 0.008	0.877 ± 0.013	0.038 ± 0.006	0.839 ± 0.014
Set: uniform frequency	0.055 ± 0.008	0.873 ± 0.013	0.051 ± 0.006	0.779 ± 0.016
Set: global frequency	0.04 ± 0.008	0.877 ± 0.013	0.039 ± 0.006	0.839 ± 0.014
Set: per-population frequency	0.04 ± 0.008	0.877 ± 0.013	0.038 ± 0.006	0.839 ± 0.015

AUCs obtained with ordered (*A*_*ord*_) and reverse (*A*_*rev*_) SNP corruption. The genotype-average baseline was selected for downstream analyses.

The genotype-average baseline emerges as a reasonable and effective choice for input baseline. It consistently achieved the best or second-best performance across all corruption methods while offering comparable results to the random, global frequency and per-population frequency baseline sets, which are more computationally demanding.

### Connecting population-genetics metrics with feature attributions

To further assess the biological relevance of the model’s attributions, we compared them to *F*_*ST*_, a well-established population-genetics metric that quantifies allele frequency differentiation between populations. By using *F*_*ST*_ as a benchmark, we explored whether the SNPs identified as important by the model align with known patterns of population structure.

**Figure 5:**
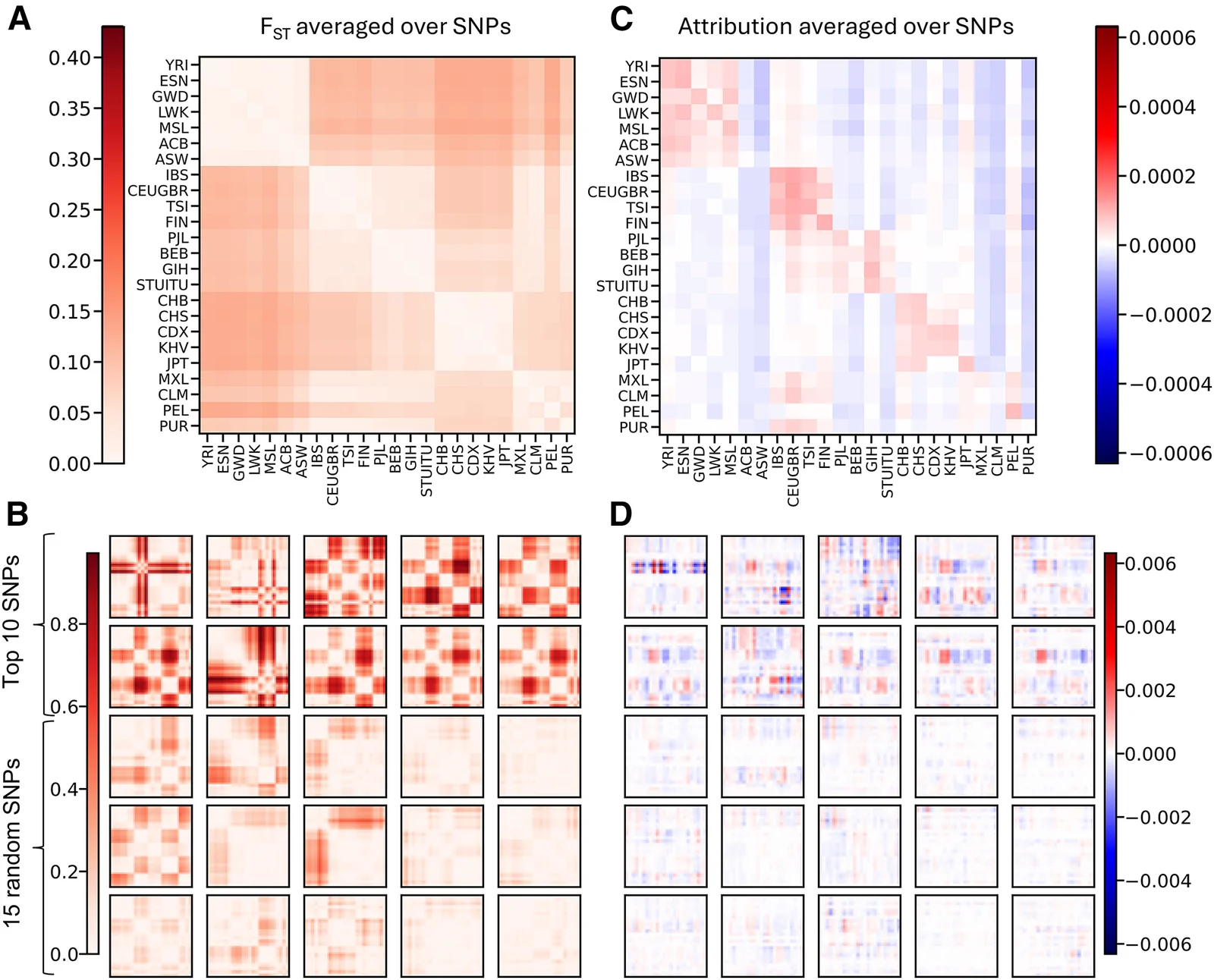
Comparing attributions with pairwise *F*_*ST*_ scores (A) 1KGP populations pairwise *F*_*ST*_ scores averaged across SNPs. (B) *F*_*ST*_ values of top 10 SNPs with the highest global attribution scores (top) and *F*_*ST*_ values of 15 randomly selected SNPs (bottom). (C) 1KGP populations pairwise attribution scores. Rows represent the true populations and columns indicate the candidate populations. The diagonal indicates the pairwise attribution score of correctly classifying individuals from a true population into the correct corresponding target population. (D) Pairwise attribution scores of the top 10 SNPs with the highest global attribution scores (top) and pairwise attribution scores of 15 randomly selected SNPs (bottom). These top and random SNPs are the same as in (B).

Population pairwise *F*_*ST*_ values, averaged across SNPs, are known to show a block structure with low genetic differentiation (*F*_*ST*_ value near 0) among populations from the same continent and higher differentiation between continents (Figure 5A). To assess whether attributions reflect population-differentiated SNPs, we computed a global importance score per SNP by averaging attribution values across individuals (see methods) and used these scores to extract the highest-ranking SNPs in the dataset. We evaluated alternative aggregation strategies for the global scores, but averaging attribution across individuals provided the most robust results for identifying model-informative SNPs (Methods S4.5; Figure S8; Table S3). Examination of *F*_*ST*_ values for the 10 SNPs with highest attribution SNP score showed stronger differentiation patterns (*F*_*ST*_ values close to 1) compared to 15 randomly selected SNPs, which displayed generally lower *F*_*ST*_ values (Figure 5B). Specifically, the 10 SNPs with the highest attribution scores fall within the top 7% of the global *F*_*ST*_ ranking (one *F*_*ST*_ value per SNP). These results suggest that the Diet Network model relies on SNPs that are differentiated between populations to make its predictions.

To explore how attribution scores reflect population structure, we computed a pairwise attribution matrix comparing actual and candidate population assignments (see methods). The resulting heatmap shows a distinct block pattern along the diagonal, indicating that SNPs tend to contribute positively to predictions within the same continental group (Figure 5C). This structure closely mirrors that of the *F*_*ST*_ matrix (Figure 5A), suggesting that the model relies on population-differentiated signals to support population prediction. Within-population attribution scores are especially elevated for most populations, further confirming that the model accumulates evidence specific to each group for correct classification.

We found that model predictions for admixed populations behave differently: for four admixed populations, ACB, ASW, CLM, and PUR, the attribution matrix showed low or negative values along the diagonal, suggesting that SNPs often reduced the probability of assigning individuals to their own population. These populations also consistently received negative attribution scores across all other groups, indicating a lack of strong positive evidence from the model. This pattern suggests that, for most populations, the Diet Network model assigns individuals by accumulating evidence based on SNPs characteristic of the population, whereas, for the admixed ACB, ASW, CLM, and PUR populations, assignment is driven by accumulating evidence against alternative populations.

When examining the pairwise attributions of the top 10 global attribution SNPs, their attribution patterns across populations mirror their *F*_*ST*_ profiles (Figure 5D). Specifically, SNPs that are not differentiated between an individual’s population and a similar target population tend to be used by the model as positive evidence for that target. Conversely, SNPs with high *F*_*ST*_ (high differentiation) relative to target populations are often used as negative evidence, reducing the probability of assignment to those targets. The similarity between the *F*_*ST*_ and attribution matrices is most evident for those SNPs with the highest attribution scores, suggesting that the model has learned to leverage population-differentiating signals in a way that reflects *F*_*ST*_-captured population structure.

## Discussion

The Diet Network is a deep-learning model designed to handle genetic data and developed in the context of genetic-ancestry prediction. Although the generalizability of the Diet Network trained on 1KGP has previously been evaluated on another population reference dataset,^36^ the HGDP dataset, our study extends this work by assessing the model on three biobanks, providing a practical assessment of generalizability, and by introducing a model explicitly trained to be robust to missing data. Furthermore, we gained insight into the model’s decision-making by applying feature-attribution methods, which confirmed that the model relied on population-differentiated SNPs. This finding is supported by a comparison with pairwise *F*_*ST*_ values, a gold-standard metric in population genetics for quantifying genetic differentiation between populations. These interpretability experiments revealed that the Diet Network adopted two main prediction strategies: for most populations, it accumulated supporting evidence, while, for specific admixed populations, it operated by eliminating alternatives, highlighting the complexity of admixed genomes. Additionally, we introduced a visualization of the model’s latent representations, enabling the characterization of individuals with admixed backgrounds not represented in the 1KGP reference panel used for training.

Several statistical and machine-learning tools have been developed to infer genetic ancestry. Some approaches, such as RFMix, Orchestra, and the support vector machine (SVM) approach developed by 23andMe,^58^ perform local-ancestry inference using haplotype-based models. These methods require phased genotype data, which not only introduces potential errors or biases but also demands considerable domain expertise and computational resources, factors that may limit scalability and broad adoption beyond specialized research settings used by population geneticists. Other methods, including admixture,^59^ infer global ancestry proportions from whole-genome data but require prior knowledge of the population composition in the dataset being analyzed to construct an appropriate reference panel. Another tool, SNVstory,^60^ adopts an ensemble strategy that combines two SVM models trained on the 1KGP and Simons Genome Diversity Project with an XGBoost model trained on the gnomAD database. This approach assigns a single population label per individual at the continental level using whole-genome data, with additional models used for finer-scale resolution within continents. However, it remains unclear how such models handle admixed individuals or generalize to new datasets with differing SNP content.

A key challenge in applying these methods across datasets lies in the variability of available genetic markers, which inevitably differ between genetic studies, biobanks, and clinical cohorts due to diverse genotyping platforms or sequencing technologies and protocols. Inference methods that rely on a fixed reference panel or ancestry-informative markers (AIMs)^61^^,^^62^ are limited by their dependence on the presence of specific predefined SNPs, reducing their applicability for datasets lacking those markers. Furthermore, deep-learning models trained on such fixed SNP sets may achieve high accuracy by over-relying on a narrow subset of highly informative variants, which can hinder generalizability.

To address these limitations, our method operates directly on unphased, unimputed genotype data, enhancing its adaptability to biobank and clinical datasets. Our training approach uses input dropout, encouraging the model to learn from a broader and more flexible set of SNPs by varying the subset it uses across samples, and mitigates overreliance on any fixed subset. As a result, the model remains robust to missing data, reducing the need for imputation and extensive preprocessing. We demonstrate that input dropout enhances the consistency of latent representations (Figure S9) and reduces prediction variability across random weight initialization (Figure S10). Moreover, unlike approaches requiring separate classifiers for different population panels, our method performs all predictions within a single unified model, simplifying deployment and improving scalability.

Despite their strong predictive performance and robustness to missing data, deep-learning models such as the Diet Network are often criticized as “black boxes,” offering limited transparency into the features driving their predictions. To address this, we applied feature-attribution methods to uncover how the model leverages genetic information. Given the computational costs of permutation-based attribution methods, we employed gradient-based approaches, which are significantly more efficient but require an appropriate input baseline as a point of comparison to quantify attributions. As most prior studies of feature attribution were developed in the context of computer vision, it remains unclear what constitutes an appropriate baseline for genetic data. We therefore designed and systematically evaluated multiple genomic baselines and found that those most effective in our setting did not align with conventional criteria used in vision models, such as neutrality and uniformity of predictions (Notes S1.3 and Figures S11 and S12). This highlights the importance of developing domain-specific evaluation metrics, such as our proposed area-based measures, to rigorously assess baseline suitability across different genomic contexts and tasks.

The attribution analyses enabled the identification of inconsistencies in the 1KGP genotyping dataset that had passed standard quality-control filters and had been used to train previous Diet Network models.^35^^,^^36^ Attribution analyses of these earlier models, trained on the genotyping dataset (3,450 samples) rather than the sequencing dataset (2,504 samples) to leverage the larger sample size, revealed SNPs with high attribution values showing unexpected divergence patterns between populations (Figure S13), consistent with observations reported in Lennon et al.^63^ These findings, together with the presence of related individuals in the genotyping dataset whose shared haplotypes could influence the patterns learned by the model, motivated the use of the sequencing dataset in the present study.

Analyzing pairwise attribution scores between populations of the models of this study provided deeper insights into the classification strategies used by the model. The distinct attribution patterns observed for ACB, ASW, CLM, and PUR (Figure 5C) of negative or near-zero diagonal attribution scores, combined with consistently negative scores across all other population columns, suggests that the model struggles to identify shared, population-specific signals likely due to high inter-individual variability in ancestral contributions. In contrast, other admixed populations, such as PEL and MXL, showed stronger within-group attribution signals, suggesting the identification by the models of population-specific variants that enable more consistent classification.

The insights gained from these interpretability analyses also shed light on the behavior of the Diet Network in cases of uncertainty. Notably, the pairwise attribution score for correctly classifying PUR individuals (Figure 5C, where both the true and target population labels are PUR) was closest to zero in comparison to the other populations. This suggests that the model assigns individuals to the PUR population not because of strong supporting evidence but rather due to the absence of strong evidence for alternative populations, effectively making PUR a default prediction in ambiguous cases. This behavior may explain why the PUR label was more frequently predicted when models were tested with high levels (99%) of missing data (Figure S5). The PUR label was also assigned by a majority of models for the input baselines containing values near the genotype mean, effectively representing missing information due to input normalization (Figure S12).

While effective in several respects described above, our approach has several limitations in its current form. First, it does not provide a built-in mechanism for estimating prediction uncertainty or confidence, a limitation in scenarios where misclassification risks must be explicitly quantified. At present, confidence could be approximated by examining the variability of model predictions for the same individual, both across multiple trained models and across the softmax probability distributions. While this approach does not yield formal confidence intervals or guarantees of correctness, as the model could consistently produce an incorrect classification, it allows for the identification of individuals whose predictions are unstable or ambiguous. In addition, our visualization of latent representations enables the detection of individuals who are genetically distinct from the training populations, as illustrated by North African individuals, who do not cluster with any existing 1KGP population in latent space (Figure 3B). This suggests that such visualizations could be used to identify out-of-distribution cases, thereby offering a complementary way to flag uncertain classifications.

Currently, the Diet Network training dataset does not include populations from several regions of the world, notably West Asia and North Africa. Future work will aim to expand population coverage beyond those represented in 1KGP to improve resolution and generalizability. While resources such as HGDP offer broader population diversity with high-quality self-reported labels, limited sample sizes constrain their direct integration into a balanced training set. Other external datasets represent valuable opportunities but often lack reliable ancestry annotations, necessitating alternative approaches such as genetic clustering, which introduce dependencies on the clustering assumptions and potential sources of errors. Finally, the use of private biobank data for training may restrict the public release of learned model parameters due to data-sharing restrictions.

At this stage, the Diet Network approach has not been implemented to support local-ancestry inference, a feature offered by tools such as RFMix^28^ that assign ancestry at a finer genomic scale. Extending it to operate across genomic windows would enable local inference and could improve resolution in admixed genomes, a feasible direction for future work. Indeed, we observed previously that Diet Network outputs trained with population-level labels correlated with RFMix-derived ancestry proportions in admixed individuals (Figure 2 in Rochefort-Boulanger et al.^36^), suggesting that the architecture can already capture aspects of admixture structure at a global level. Building on this foundation, a window-based extension could further enhance resolution. Haplotype-level information could further enhance prediction accuracy by capturing linkage patterns that single SNPs may miss, although it would require rethinking the input representation or adapting the Diet Network to integrate hierarchical transformer architectures, which are well suited for modeling haplotype-level information and long-range dependencies between genetic regions.^64^ Such extensions would likely improve the characterization of admixed populations by capturing differences in ancestral composition and segment-level structure, thereby helping to distinguish between admixed groups that share the same source populations but differ in their genomic ancestry patterns. This approach could also facilitate generalization to admixed populations not explicitly represented in the training set, provided their ancestral source populations are included during training.

Accurate genetic-ancestry prediction is essential for promoting equity in biomedical research and fairness in artificial intelligence (AI) tools for health. There is broad consensus on the need to develop predictive tools that are generalizable, unbiased, and inclusive of global population diversity, but this effort depends on how ancestry is identified and annotated in health studies. Our method supports this goal by democratizing ancestry annotation within biobanks, including for individuals with missing data, by refining broad categories into more granular population groups. Additionally, the method can facilitate the identification of individuals from local biobanks who are suitable for inclusion in population reference panels, thus leveraging the rich, region-specific genetic diversity available in these resources. As such, the Diet Network represents a step toward a more equitable foundation for AI development in health applications, supporting better assessment of model performance across ancestries in genomic research and ultimately supporting the design of fairer, more inclusive AI systems.

## Data and code availability

The Diet Network code generated during this study is available on GitHub: https://github.com/HussinLab/DIETNETWORK. This includes scripts for performing inference with trained models on new datasets as well as for training models from scratch. The trained model weights along with the files required for inference, and the 1KGP dataset used for training, are available on Zenodo: https://doi.org/10.5281/zenodo.16943452.

## Acknowledgments

We thank Yoshua Bengio, Marc-André Legault, and Olivier Tastet for their valuable discussions and insights regarding the experiments and results as well as Léo Choinière and Vanessa Bellegarde for their contributions, which helped advance our work. We also thank the participants of the 1KGP, the CaG biobank, the MHIB, and the AoU biobank for their contributions, which have made this research possible. This work was made possible through computational resources provided by the Digital Research Alliance of Canada.

This study was supported by funding from the 10.13039/501100012651Montreal Heart Institute Foundation, an 10.13039/501100019217IVADO PRF grant to J.G.H. (PRF-2019-3378524797) and a 10.13039/501100000038Natural Sciences and Engineering Research Council of Canada (NSERC) Discovery grant to J.G.H. (RGPIN-2022-04262). C.R.-B. and M.S. were supported by a Canada Graduate Scholarship from NSERC. J.G.H. holds a Tier 2 Canadian Research Chair from the NSERC in Responsible Multi-omics Data Science.

## Author contributions

C.R.-B. and M.S., conceptualization, formal analysis, investigation, methodology, software, validation, visualization, and writing – original draft and review & editing; R.P. and J.-C.G., data curation, formal analysis, methodology, validation, visualization, and writing – review and editing; P.L.C., validation and writing – review and editing; S.L., supervision, validation, and writing – review and editing; J.G.H., conceptualization, funding acquisition, project administration, resources, supervision, validation, and writing – review and editing.

## Declaration of interests

The authors declare no competing interests.

## Declaration of generative AI and AI-assisted technologies in the manuscript preparation process

During the preparation of this work, the authors used ChatGPT (OpenAI) in order to improve the readability and language of sections of the manuscript. After using these tools, the authors reviewed and edited the content as needed and take full responsibility for the content of the published article.

## Supplemental information

Document S1. Figures S1–S13, Tables S1–S3, supplemental notes, and supplemental methods

Document S2. Article plus supplemental information
