# Supplementary material for: A transparent and generalizable deep-learning framework for genomic ancestry prediction
Source: Am J Hum Genet. 2026 Jul 2;113(7):1461–77. doi: 10.1016/j.ajhg.2026.06.003 (PMC13504326; doi:10.1016/j.ajhg.2026.06.003)
Supplement: Supplementary file 1 [file mmc1.pdf]

The American Journal of Human Genetics, Volume *113*

Supplemental information

A transparent and generalizable deep-learning framework for genomic ancestry prediction

Camille Rochefort-Boulanger, Matthew Scicluna, Raphaël Poujol, Jean-Christophe Grenier, Pierre Luc Carrier, Sébastien Lemieux, and Julie G. Hussin

S1 Supplemental Notes

S1.1 Population label merging in 1KGP dataset

The decision to merge specific ancestry labels was guided by a combination of quantitative analyses and empirical observations.

We first examined pairwise F_{ST} values [1, 2] across all population pairs and reported, for each population, the minimum F_{ST} observed with any other population (Figure S2A). Several pairs exhibited low genetic differentiation, including CEU–GBR, ESN–YRI, CHS–CHB, and ITU–STU. However, low F_{ST} alone was not used as a merging criterion, as some pairs with small F_{ST} values (e.g., ESN–YRI and CHB–CHS) were still distinguishable by our models.

We therefore complemented the F_{ST} analysis with PCA, focusing on population pairs with low genetic differentiation: CEU-GBR, ESN-YRI, CHB-CHS, and ITU-STU. Our criterion was that if two populations could be separated along the first two principal components, this indicated meaningful structure and argued against merging. ESN–YRI and CHB–CHS remained separable (Figure S2B), whereas CEU–GBR showed substantial overlap. For the ITU–STU pair, we observed internal substructure within ITU, including a subgroup clustering closely with STU.

Finally, we considered evidence from prior literature. Previous work developing ancestry inference methods has merged CEU and GBR [3]. For ITU and STU, prior studies reported within-population substructure and genetic similarity across these groups [4], including the identification of related individuals across the two populations [5]. Taken together, these analyses supported merging CEU–GBR and ITU–STU while retaining other closely related population pairs as distinct labels.

S1.2 Grand-parental information of MHIB Africans

Of the 117 individuals with self-reported African ancestry in MHIB, grand-parental country of birth data were available for 58, with 46 reporting at least one grandparent born in Haiti. This suggests that a substantial proportion of self-reported African individuals in MHIB may be from Haiti, which could explain the consistency in model predictions observed between the CaG and MHIB cohorts. Furthermore, compared to the prediction for Haitians in CaG, there was broader representation of other African populations (e.g., LWK, GWD, ESN, MSL) in MHIB.

S1.3 Investigating baselines neutrality

We evaluated whether our genomics-informed baseline inputs satisfy prediction neutrality, the expectation that model predictions for baseline inputs should not be biased toward specific classes (populations). This assessment was conducted for both single-sample baselines and sets of baselines using two metrics: (1) the entropy of the predicted probability distributions returned by the models, which reflects the uncertainty of the predictions, and (2) the variability of predicted classes across models, which indicates how evenly predictions are distributed across categories. We assessed the consistency between the best-performing baselines identified using the A_{ord} and A_{rev} metrics (i.e., the genotype-average single baseline

and the multi-sample baselines: random sets, global set, and per-population set) and those characterized as more neutral based on our entropy and class diversity criteria.

S1.3.1 Entropy

Among all baselines, the Highest-entropy single-sample baseline and the Uniform frequency baseline set exhibited the highest prediction entropy (Figure S11), despite being among the worst performers based on the area metrics (Table 2). Conversely, the random samples and per-population set baselines, which achieved high performance according to the area metrics, displayed the lowest prediction entropy. This suggests that prediction neutrality, as measured by entropy, does not reliably distinguish between effective and ineffective baselines in our genomic context.

S1.3.2 Prediction variability

Investigating the prediction variability revealed that the per-population baseline set, one of the top performers based on the area metrics (Table 2), was the only baseline to produce a balanced distribution of predicted classes across all populations (Figure S12). In contrast, other high-performing baselines, such as the Genotype-average and Global frequency set exhibited prediction biases toward specific populations, particularly PUR. This indicates that prediction neutrality, as measured by the variability in the predicted classes, does not consistently identify effective baselines.

Overall, the assessment of baseline neutrality using entropy and predicted class variability does not provide reliable guidance for determining the suitability of baselines in our genomic context, emphasizing the importance of incorporating our area metrics in the evaluation process.

S2 Supplemental Figures

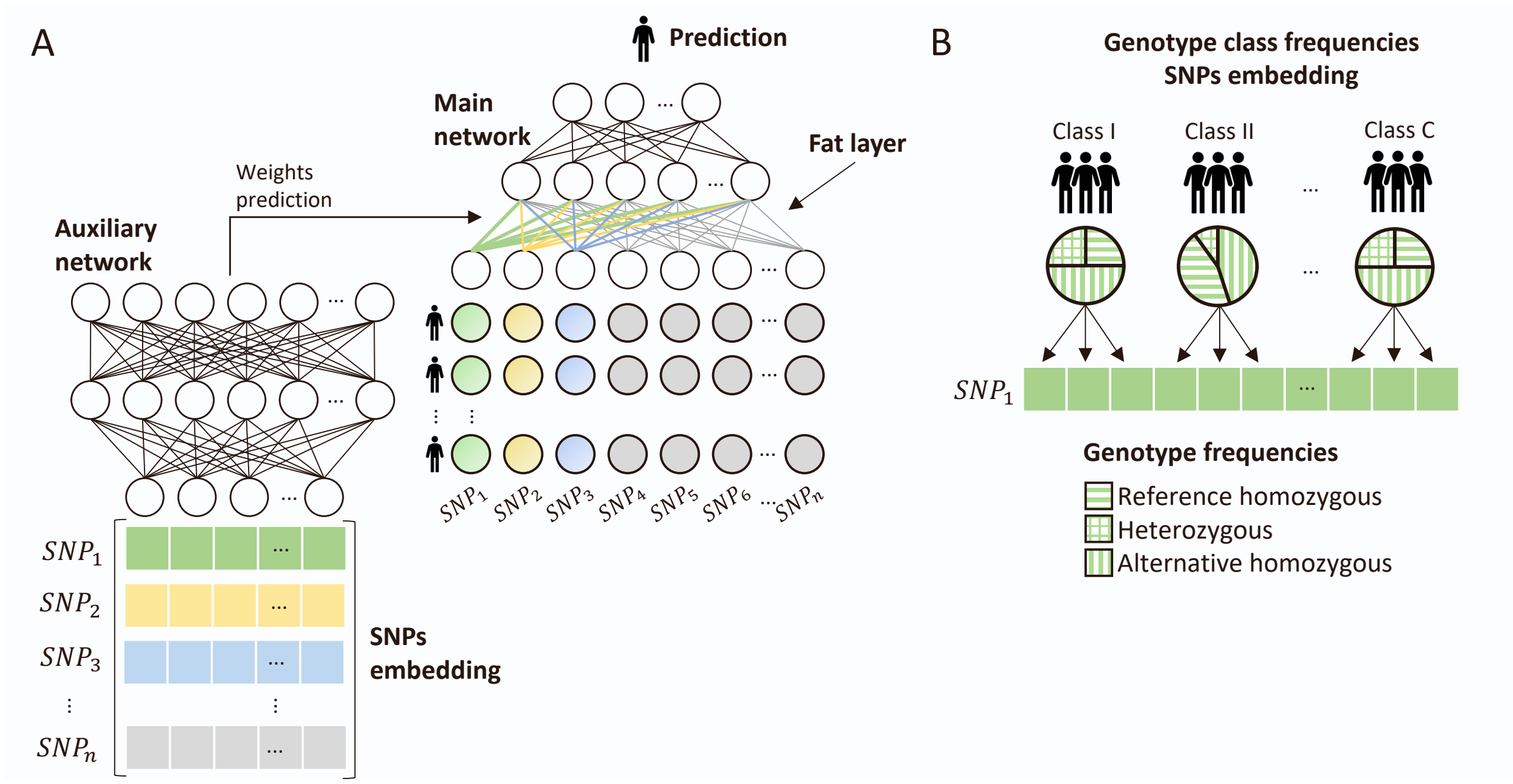

Figure S1: Diet Network architecture. A) The Diet Network is composed of two neural networks trained jointly: a main network and an auxiliary network. The main network takes as input individual-level genotype data, encoded additively as 0, 1, or 2, representing the number of alternative alleles relative to the reference genome. The final output of the main network is a 24-dimensional vector corresponding to the 24 populations in 1KGP (Supplemental Notes **S1.1**), obtained after a softmax transformation to produce the Diet Network scores. The predicted population corresponds to the label with the highest Diet Network score. Due to the high dimensionality of SNP data, this input generates a large number of weights in the initial layer, referred to as the *fat layer*. To reduce the number of trainable parameters and mitigate overfitting, the auxiliary network learns to predict the weights of this fat layer for each SNP. The auxiliary network receives as input a representation of each SNP, called the *SNPs embedding*. **B)** Example of the input provided to the auxiliary network for a single SNP (SNP_1). In our population classification task, SNP embeddings are computed from the genotype frequencies across populations. The input vector is constructed by concatenating the frequencies of each genotype (0, 1, 2) across the 24 populations from the 1KGP, resulting in a 72-dimensional vector ($24 \times 3 = 72$). Genotype frequencies are calculated using the training set within each cross-validation fold.

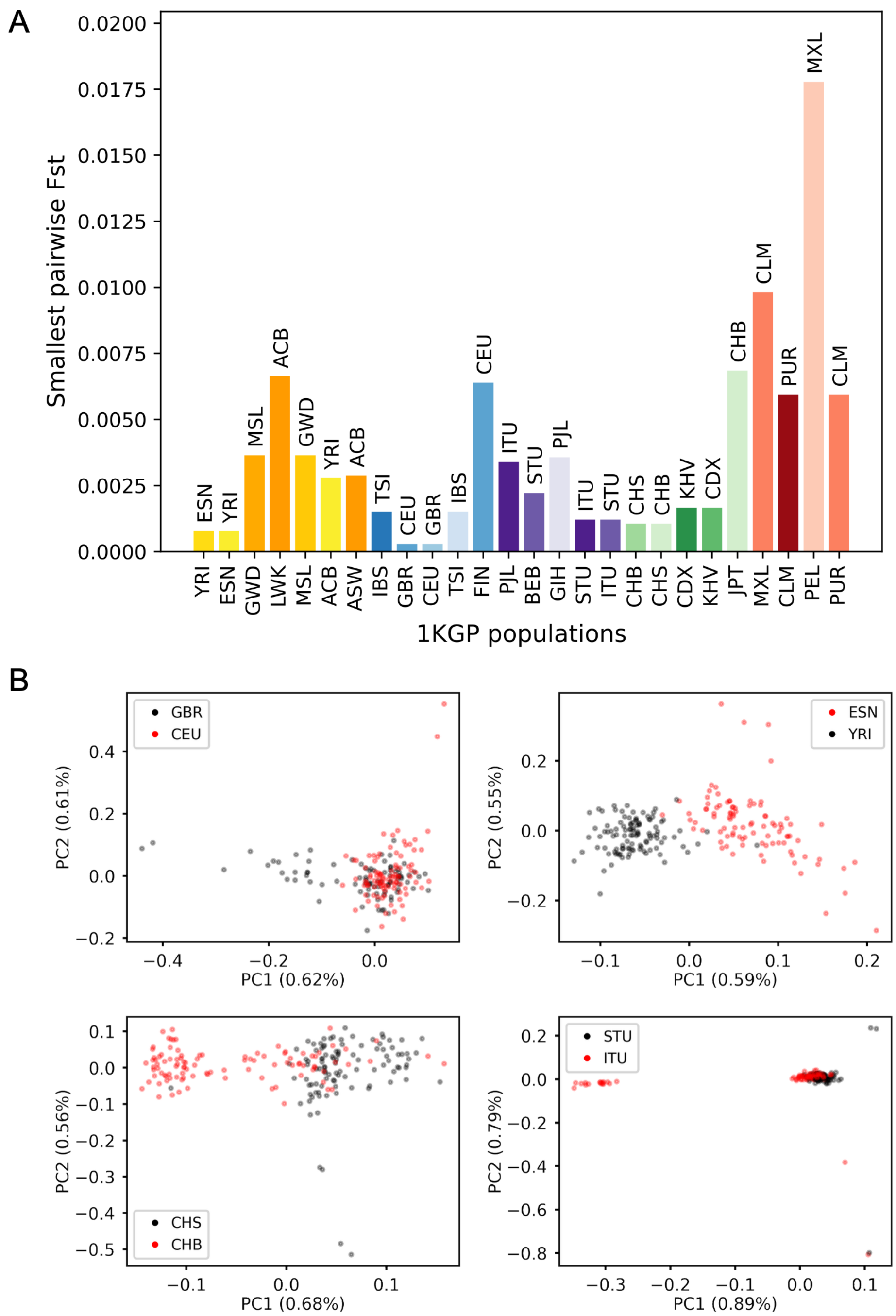

Figure S2: Pairwise F_{ST} and PCA of closely related 1KGP populations (A) Pairwise F_{ST} was computed for each 1KGP population pair. For each population (x-axis), the y-axis shows the smallest F_{ST} observed with any other population. The corresponding population with the smallest F_{ST} value is indicated above each bar. (B) PCA of population pairs with low F_{ST}. PCA was performed using PLINK on population pairs exhibiting low pairwise F_{ST} and outlier individuals were removed. Each point represents an individual and is colored according to its population label.

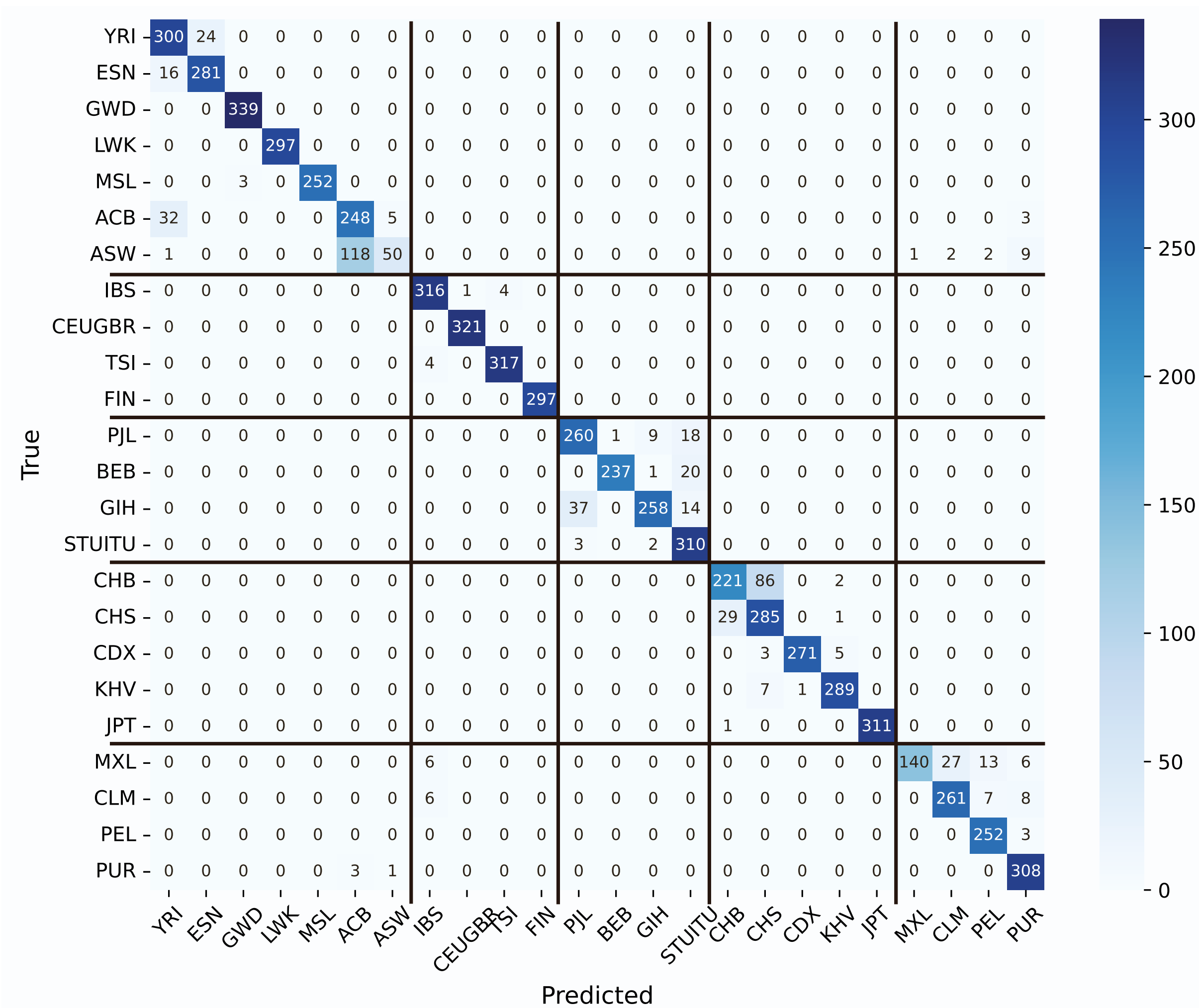

Figure S3: Diet Network models predictions on 1KGP test sets. Population labels predicted by fifteen Diet Networks models trained using 5-folds cross-validation across three random seeds, with an input dropout rate of 0.995. Black lines outline 1KGP super-population. The confusion matrix displays predictions on the held-out test set from each fold. As each individual appears once in the test set per cross-validation run and the procedure is repeated across three random seeds, each individual contributes three predictions to the confusion matrix.

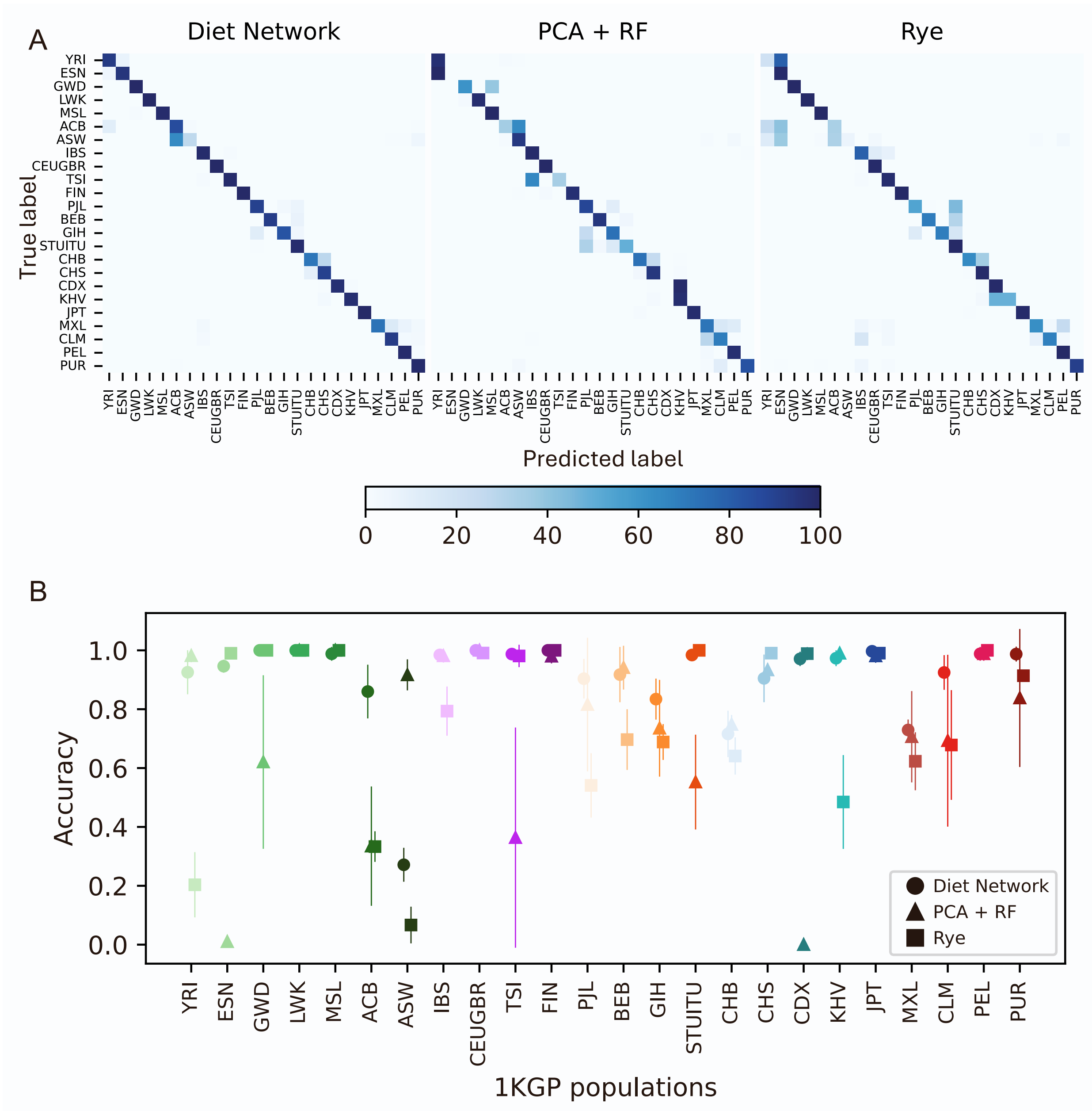

Figure S4: Performance comparison of Diet Network, PCA+RF and Rye methods. (A) Confusion matrices of each method. Color scale indicates the percentage of classified samples for each population. (B) Per population classification accuracy of the three methods, computed as the proportion of correctly classified individuals within each population. Error bars represent de standard deviation across the five folds.

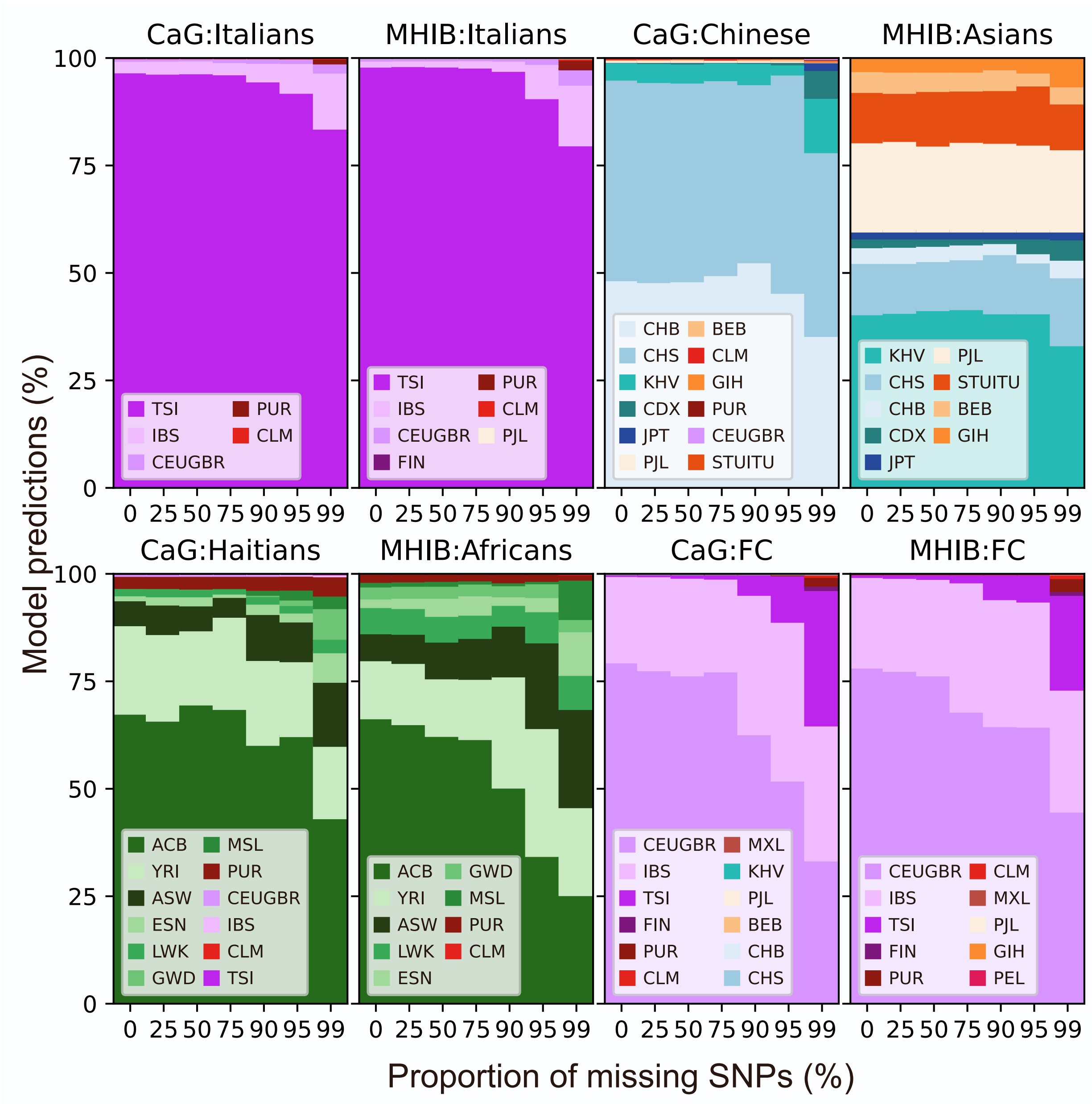

Figure S5: Generalizability on CaG and MHIB of Diet Network models trained with 0.995 input dropout across proportions of missing SNPs. 1KGP predicted populations for each of selected proxy populations in CaG GSA subset and MHIB. Each column represents the proportion of SNPs removed to simulate missingness, and within each column the distribution of model predictions across all individuals is shown. FC denotes French Canadian individuals.

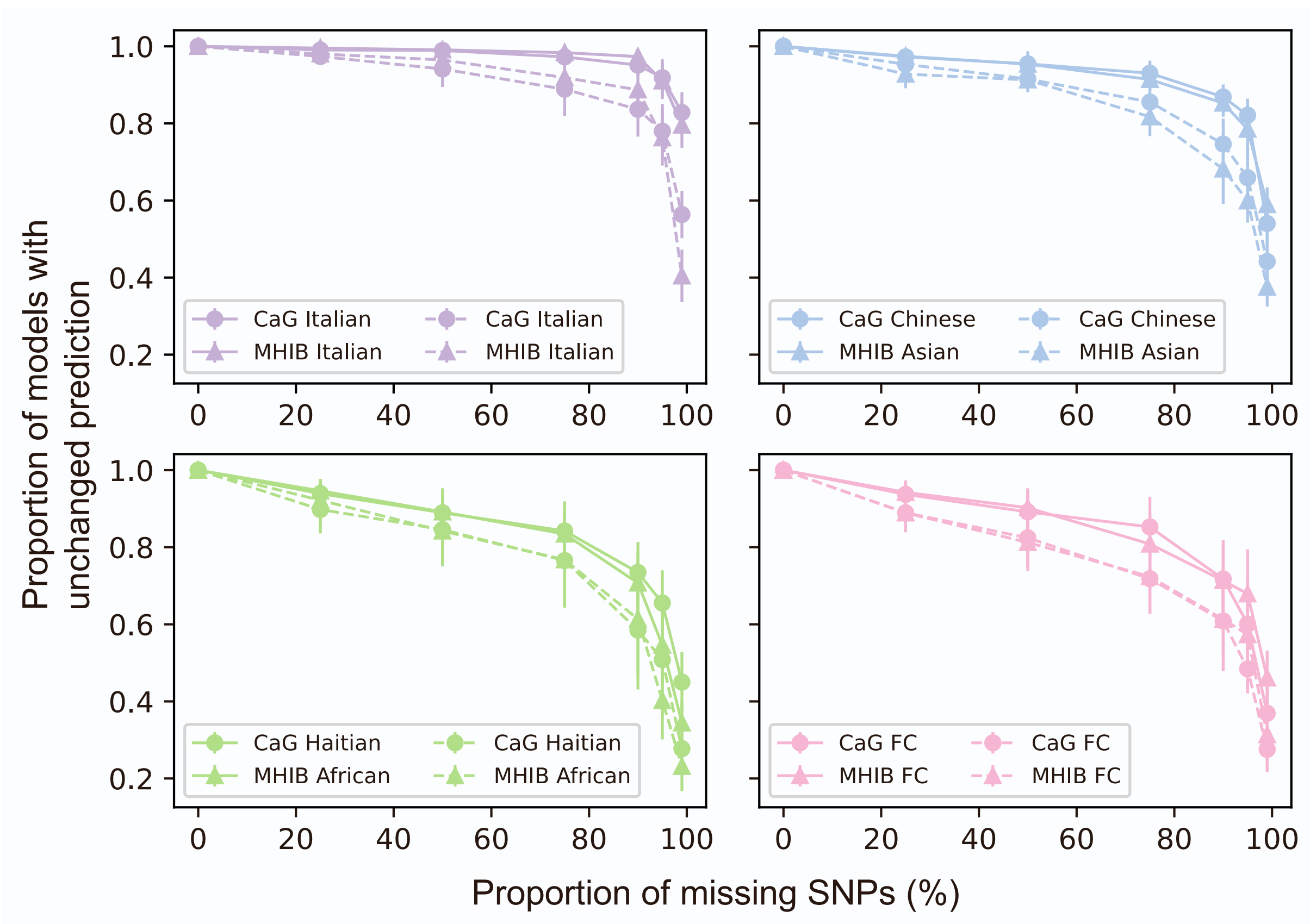

Figure S6: Input dropout impact on prediction changed across missing rates. Proportion of models with an unchanged prediction across missing rates compared to predictions on complete SNPs data. Error bars represent the standard deviation across individuals in each CaG GSA and MHIB population. Dashed lines correspond to predictions of models trained without input dropout, while solid lines indicate predictions of models trained with an input dropout rate of 0.995. FC denotes French Canadian individuals.

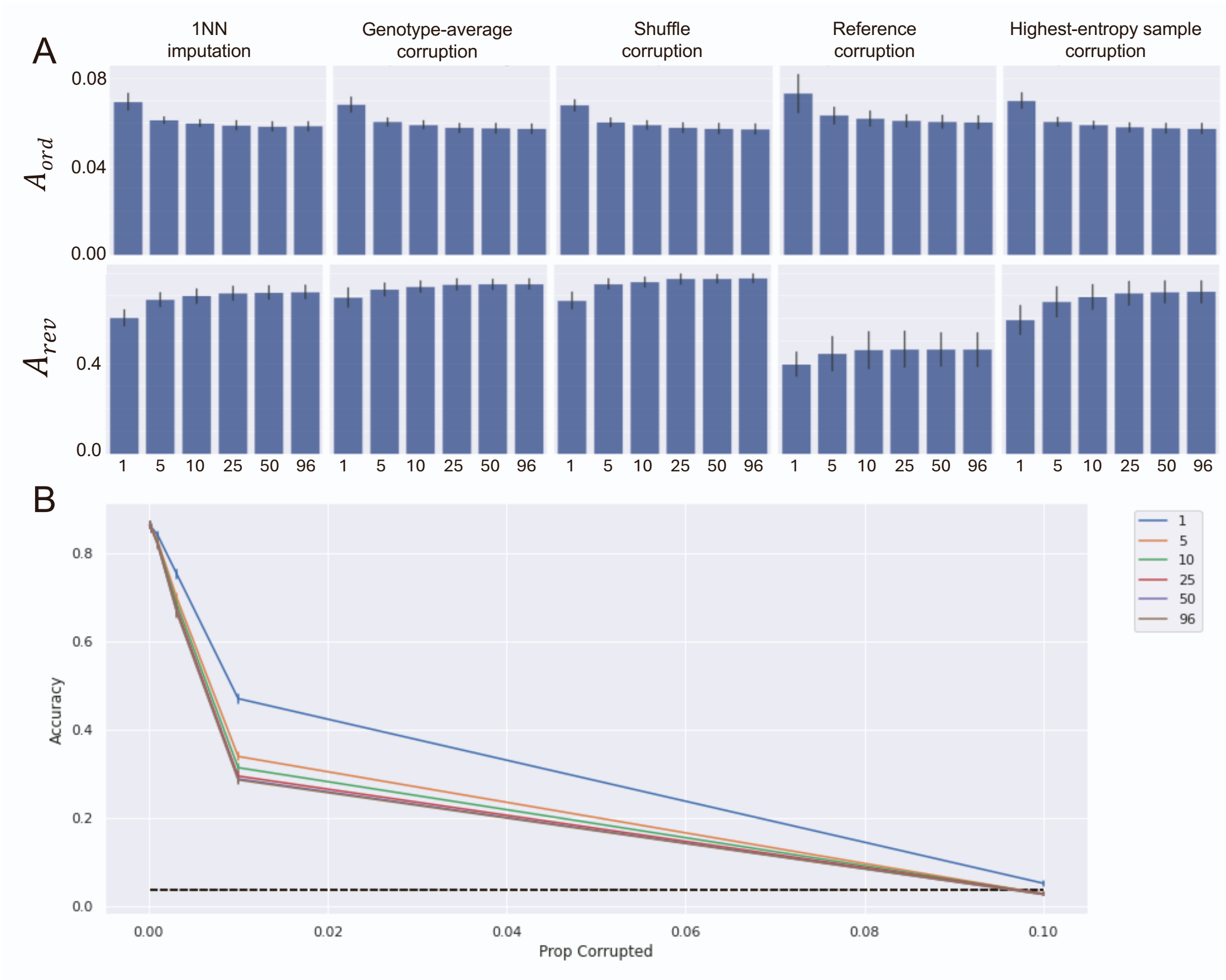

Figure S7: Area metrics for suboptimal baselines. Suboptimal baseline sets were generated by sampling subsets of 1, 5, 10, 25, and 50 individuals from the full 96-sample multi-sample baseline. Attribution scores were computed using Integrated Gradients on fifteen Diet Network models trained via 5-fold cross-validation across three random seeds with 99.25% input dropout rate. (A) shows A_{ord} and A_{rev} metrics, where the single-sample baseline consistently performs worst. (B) shows classification accuracy for proportion of corrupted SNPs. A faster decline in accuracy is observed as the number of random samples included in the baseline set increases, reflecting the poorer ability of suboptimal baselines to identify important SNPs. Error bars represent the standard deviation across each of the 15 models. Legend indicates the number of random samples in each baseline set. We show only up to 10% corruption for visual clarity.

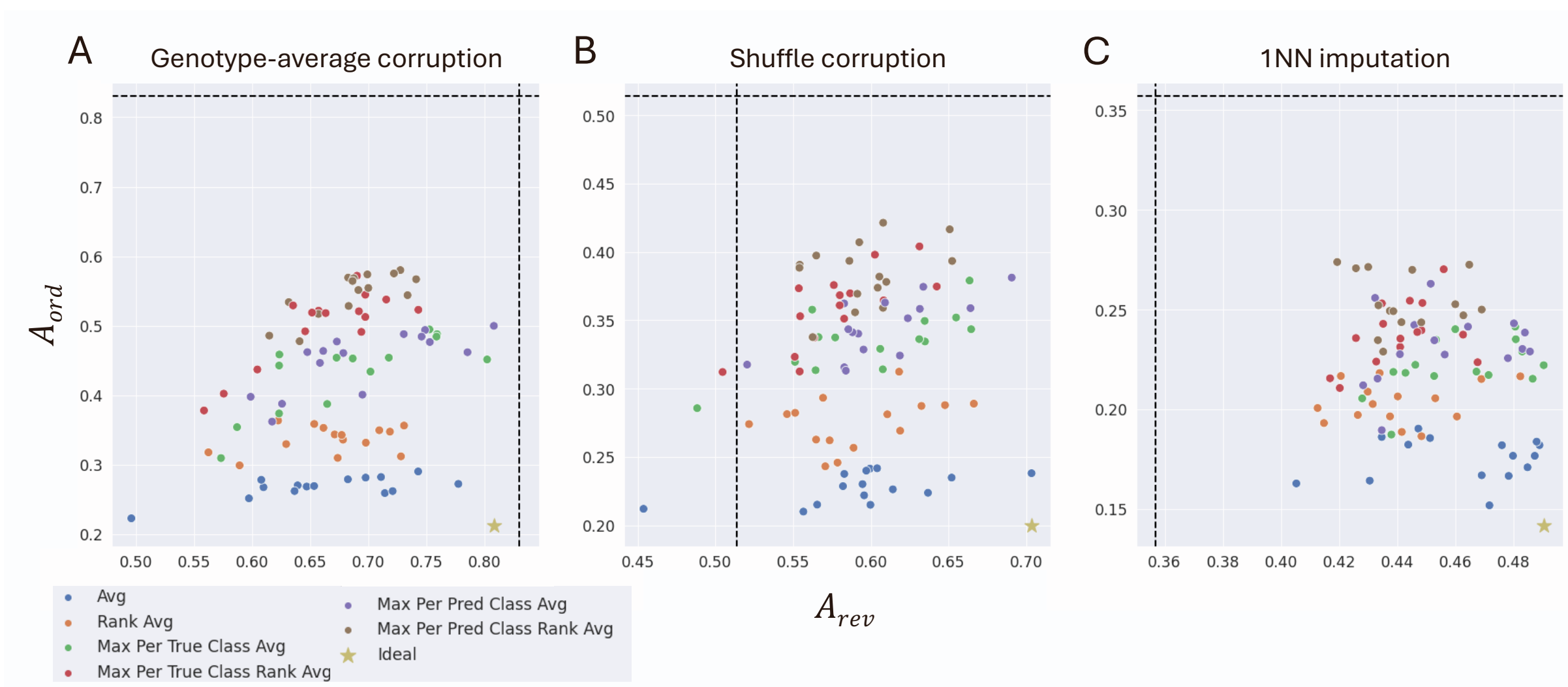

Figure S8: Global score aggregation strategies performance. A_{ord} (y-axis) and A_{rev} (x-axis) computed for SNPs exclusion experiments performed using global scores computed for different local scores aggregation strategies. Local attribution scores were computed using Integrated Gradients and the genotype-average baseline on fifteen models trained with 99.25% input dropout. We compare the models' area metrics against an ideal score (designated by a star in the bottom right corner). Horizontal and vertical lines represent A_{ran}, the area under the curve obtained when corrupting SNPs at random. SNPs were corrupted using (A) genotype-average corruption, (B) shuffle corruption and (C) 1NN imputation.

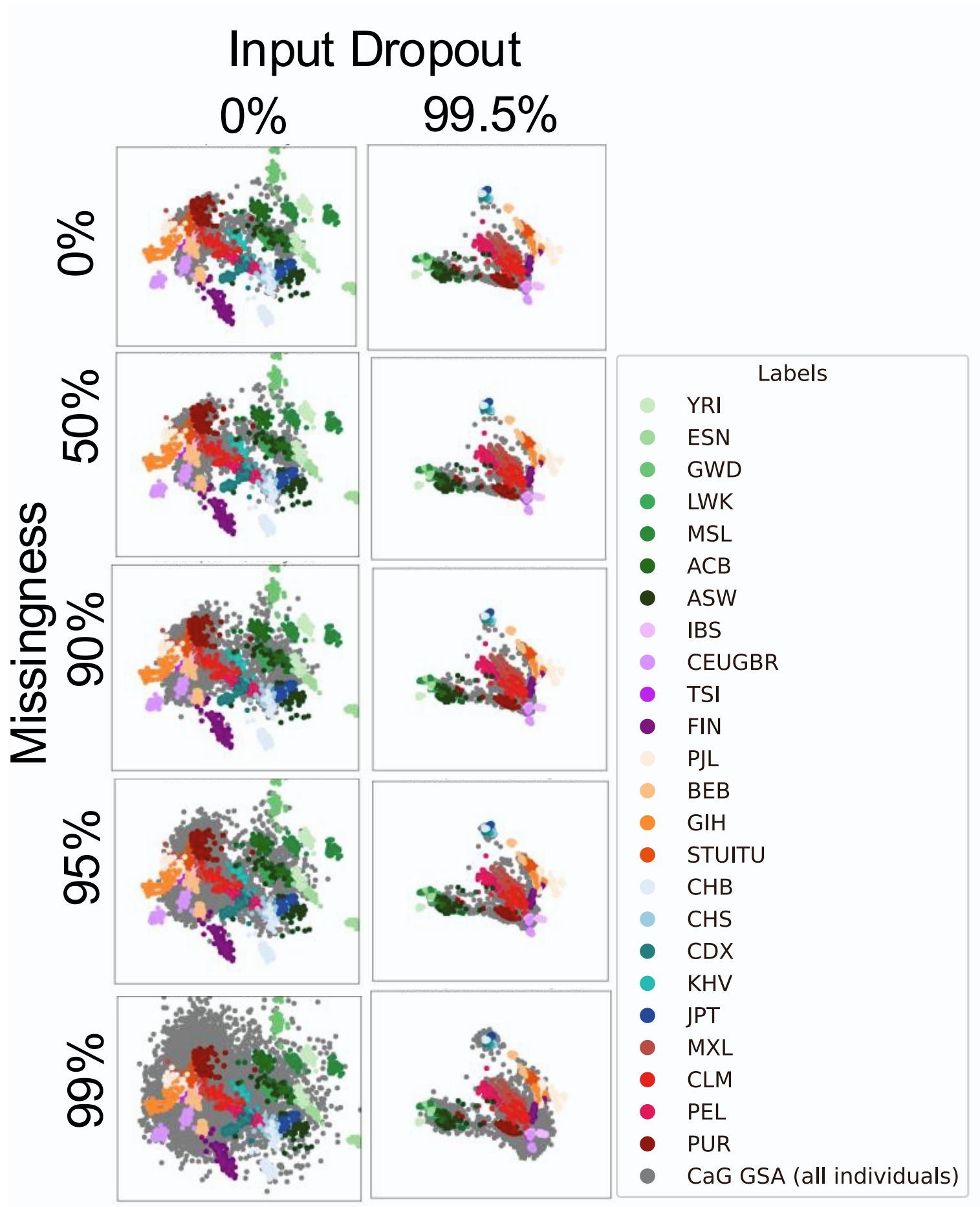

Figure S9: Diet Network model trained with input dropout yields more consistent hidden layer representations across different levels of missing SNPs. PCA of values in the last hidden layer (100 neurons) of Diet Network models trained on 1KGP. The PCA was computed using 1KGP individuals from the test set and used to project the last hidden layer representations of all 17,286 individuals in the CaG GSA subset. The plots show results for models trained without input dropout (left) and with an input dropout rate of 0.995 (right). Each row represents a different level of missingness, corresponding to the number of SNPs removed in the CaG dataset.

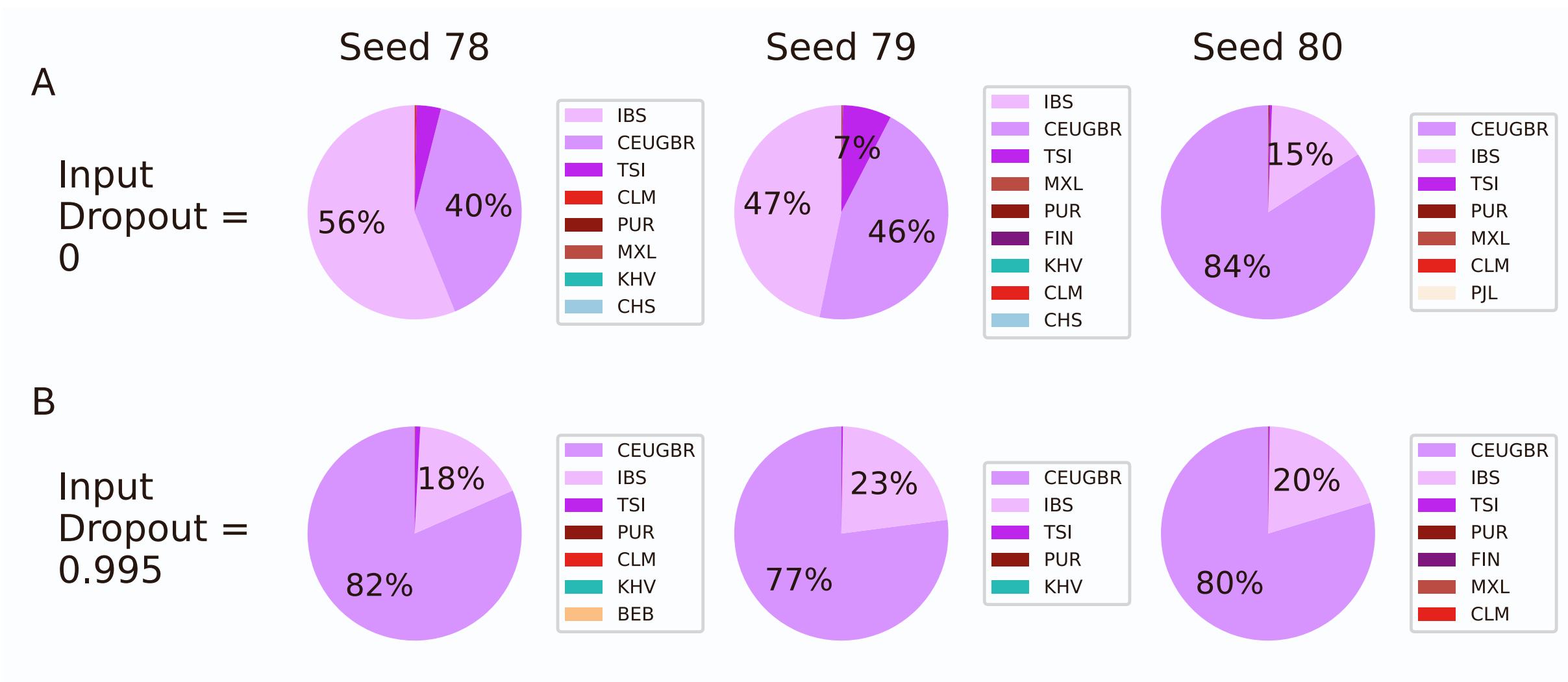

Figure S10: Diet Network models trained with input dropout yield more consistent predictions across random seeds. Diet Network models predictions for CaG French Canadian individuals across three random seeds. Each pie chart represent predictions of 5 models (from the 5-folds cross-validation) for a given seed. (A) Predictions of Diet Network models trained without input dropout (B) Predictions of Diet Network models trained with a 0.995 input dropout rate.

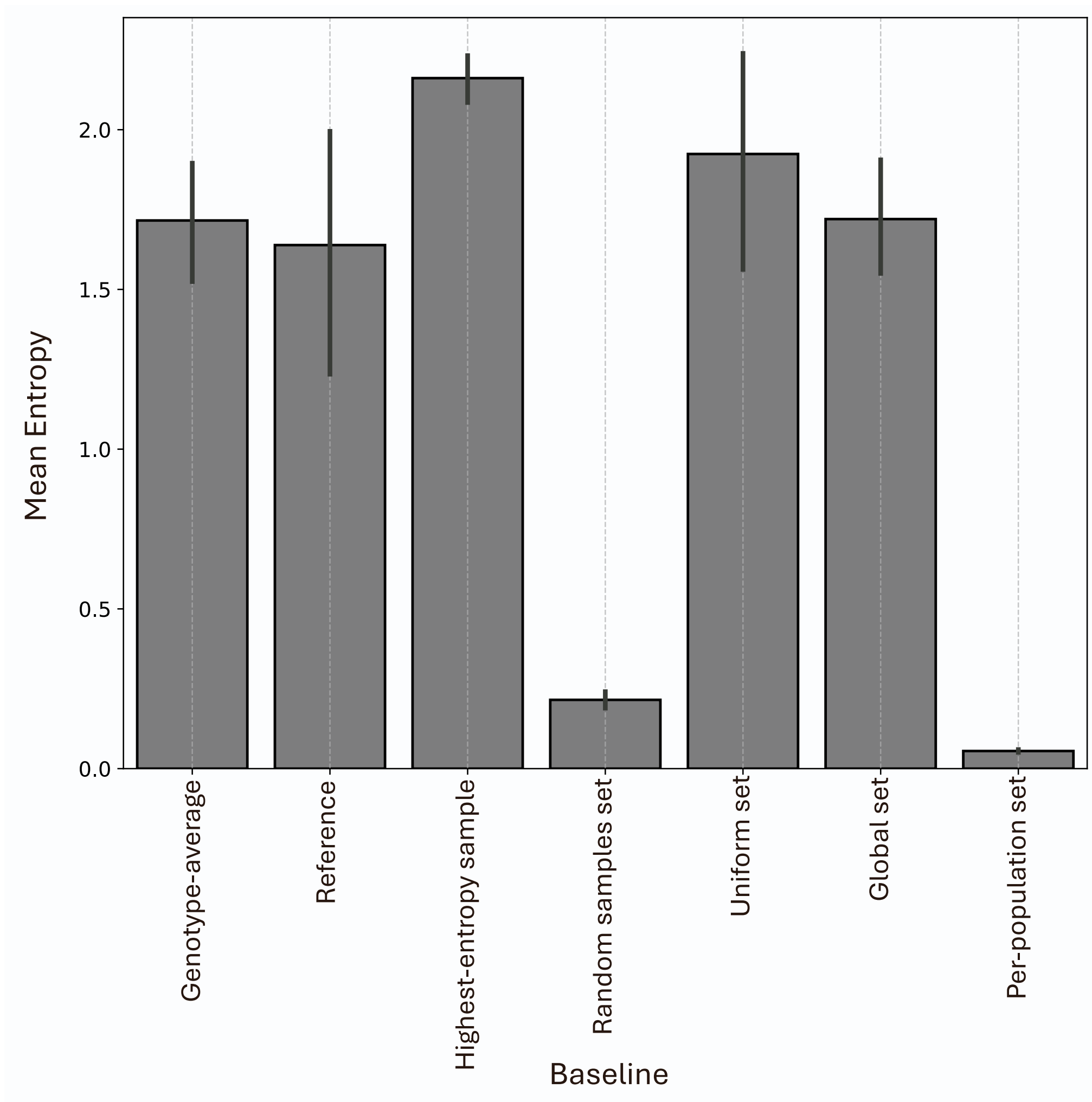

Figure S11: Prediction entropy of baselines. Prediction entropy (y-axis) computed for each baseline input (x-axis) using fifteen models trained with 99.5% input dropout. Error bars represent the standard deviation across each of the 15 models. For multi-sample sets, prediction entropy for all baselines in the set were counted.

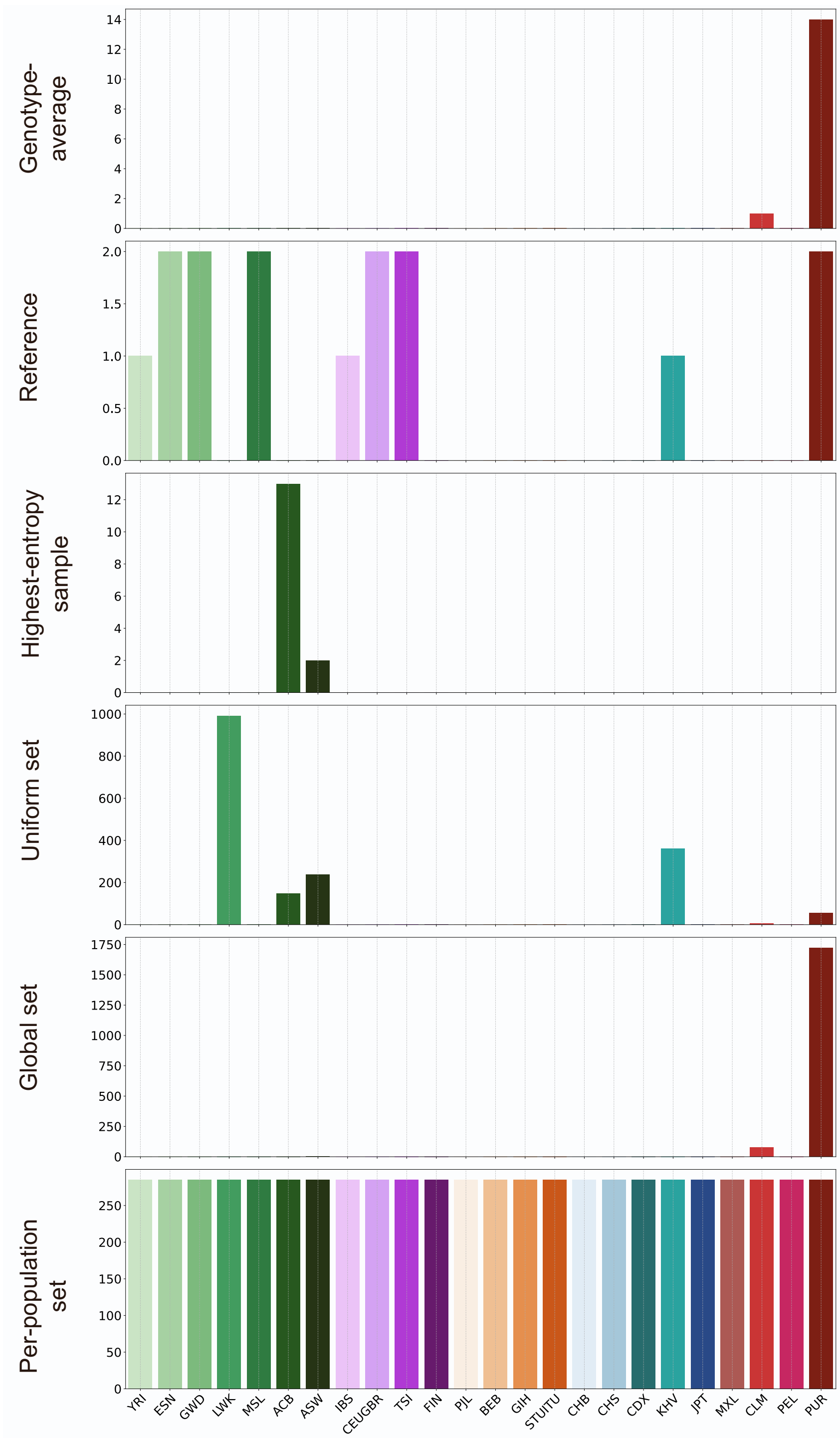

Figure S12: Prediction Diversity per Baseline. Number of predicted classes (1KGP populations) for each baseline by the fifteen models trained with 99.5% input dropout. For multi-sample sets, we count predicted classes for all baselines in the set (15 models × 96 baselines per set).

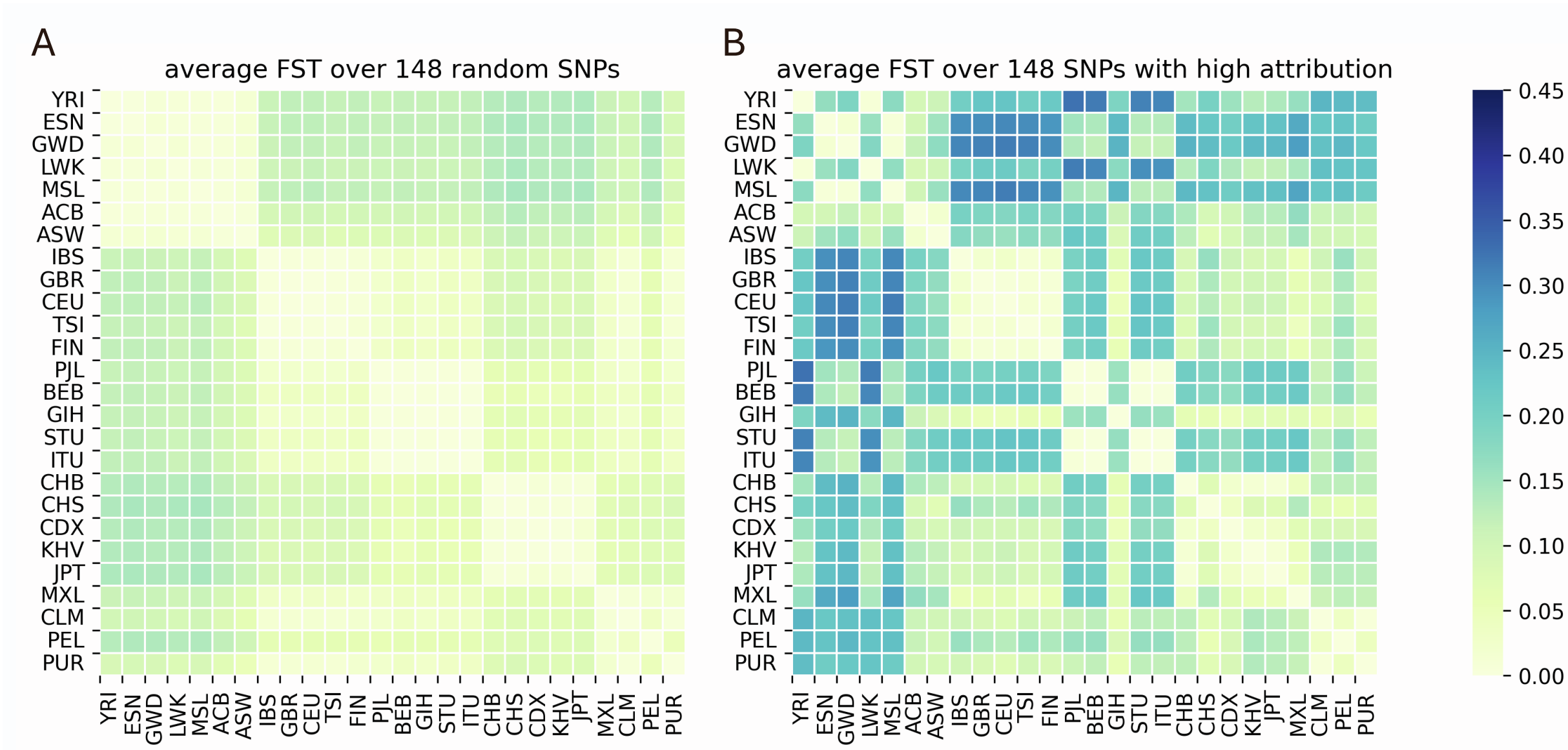

Figure S13: Population pairwise F_{ST} of random and highly attributed SNPs. Pairwise F_{ST} values computed between all 1KGP population pairs and averaged across SNPs using data from the 1KGP Phase 3 dataset which includes 3,450 individuals genotyped in batches using the Affymetrix genotyping array. SNPs were filtered following the steps outlined in the Methods section, yielding 294,427 SNPs. (A) Average pairwise F_{ST} of 148 randomly selected SNPs shows typical population structure patterns, with populations within continental groups having lower F_{ST} values. (B) Average pairwise F_{ST} for the 148 SNPs with the highest attribution scores identified using the Integrated Gradients method applied to fifteen Diet Network models trained via 5-fold cross-validation with three random seeds. The F_{ST} patterns of those high attribution SNPs exhibit unexpected divergence. Notably, unusually high differentiation was observed between pairs of closely related populations including YRI with ESN, GWD, and MSL, and GIH with PJL, BEB, ITU, and STU. These SNPs showed unexpected divergence, including high differentiation between closely related populations (e.g., YRI vs. ESN/GWD/MSL; GIH vs. PJL/BEB/ITU/STU). Comparing 1KGP genotyping array data to the 1KGP high-coverage whole genome sequencing data revealed allele frequency discrepancies greater than 20%, suggesting genotyping errors not removed by standard filters.

S3 Supplemental Tables

Table S1: Thousand Genomes (1KG) Project Populations Code and Description. Officially approved population descriptors were established to accurately represent both the ancestral geography or ethnicity of each population and the geographic location where the samples were collected. These descriptors incorporate input from sample donor communities regarding how they wish to be described and should be used as designated without modification.

Code	Superpopulation code	Description
YRI	AFR	Yoruba in Ibadan, Nigeria
ESN	AFR	Esan in Nigeria
GWD	AFR	Gambian in Western Division, The Gambia - Mandinka
LWK	AFR	Luhya in Webuye, Kenya
MSL	AFR	Mende in Sierra Leone
ACB	AFR	African Caribbean in Barbados
ASW	AFR	African Ancestry in Southwest US
IBS	EUR	Iberian populations in Spain
CEU	EUR	Utah residents (CEPH) with Northern and Western European ancestry
GBR	EUR	British in England and Scotland
TSI	EUR	Toscani in Italy
FIN	EUR	Finnish in Finland
PJL	SAS	Punjabi in Lahore, Pakistan
BEB	SAS	Bengali in Bangladesh
GIH	SAS	Gujarati Indians in Houston, TX
STU	SAS	Sri Lankan Tamil in the UK
ITU	SAS	Indian Telugu in the UK
CHB	EAS	Han Chinese in Beijing, China
CHS	EAS	Han Chinese South
CDX	EAS	Chinese Dai in Xishuangbanna, China
KHV	EAS	Kinh in Ho Chi Minh City, Vietnam
JPT	EAS	Japanese in Tokyo, Japan
MXL	AMR	Mexican Ancestry in Los Angeles, California
CLM	AMR	Colombian in Medellin, Colombia
PEL	AMR	Peruvian in Lima, Peru
PUR	AMR	Puerto Rican in Puerto Rico

Codes and descriptions are provided by the International Genome Sample Resource [6] and listed in the order used in the paper figures. Superpopulation codes: African ancestry (AFR), Americas (AMR), East Asian ancestry (EAS), European ancestry (EUR), South Asian ancestry (SAS)

Table S2: Area metrics across baselines for corruption methods that replace corrupted SNPs with corresponding values found in single-sample baselines

	Reference Corruption		Genotype-average Corruption		Highest-entropy sample Corruption	
**Baseline**	A_{ord}	A_{rev}	A_{ord}	A_{rev}	A_{ord}	A_{rev}
Reference	0.02 ± 0.01	0.85 ± 0.03	0.05 ± 0.01	0.92 ± 0.01	0.04 ± 0.01	0.79 ± 0.04
Genotype-average	0.03 ± 0.01	0.8 ± 0.05	0.04 ± 0.01	0.92 ± 0.01	0.04 ± 0.01	0.87 ± 0.02
Highest-entropy sample	0.04 ± 0.01	0.77 ± 0.07	0.05 ± 0.01	0.92 ± 0.01	0.03 ± 0.01	0.88 ± 0.01
Set - Random samples	0.03 ± 0.01	0.8 ± 0.05	0.04 ± 0.01	0.92 ± 0.01	0.04 ± 0.01	0.87 ± 0.02
Set - Uniform frequency	0.06 ± 0.01	0.73 ± 0.06	0.05 ± 0.01	0.92 ± 0.01	0.05 ± 0.01	0.82 ± 0.03
Set - Global frequency	0.03 ± 0.01	0.8 ± 0.05	0.04 ± 0.01	0.92 ± 0.01	0.04 ± 0.01	0.87 ± 0.02
Set - Per-population frequency	0.03 ± 0.01	0.8 ± 0.05	0.04 ± 0.01	0.92 ± 0.01	0.04 ± 0.01	0.87 ± 0.02

Table S3: Area metrics for corruption order based on SNPs global attribution scores.

Corruption Style	Score	A_{ord}	A_{rev}
Genotype-average	Avg	0.2679 ± 0.0160	0.6621 ± 0.0703
	Rank Avg	0.3368 ± 0.0196	0.6667 ± 0.0494
	Max Per True Class Avg	0.4329 ± 0.0530	0.6821 ± 0.0680
	Max Per True Class Rank Avg	0.5000 ± 0.0536	0.6612 ± 0.0516
	Max Per Pred Class Avg	0.4509 ± 0.0429	0.6950 ± 0.0637
	Max Per Pred Class Rank Avg	0.5460 ± 0.0320	0.6865 ± 0.0375
	Random SNP Corruption	0.8300 ± 0.0174	
Shuffle	Avg	0.2277 ± 0.0111	0.5958 ± 0.0533
	Rank Avg	0.2752 ± 0.0188	0.5905 ± 0.0405
	Max Per True Class Avg	0.3334 ± 0.0226	0.5973 ± 0.0506
	Max Per True Class Rank Avg	0.3586 ± 0.0277	0.5780 ± 0.0345
	Max Per Pred Class Avg	0.3449 ± 0.0217	0.6068 ± 0.0400
	Max Per Pred Class Rank Avg	0.3842 ± 0.0229	0.5955 ± 0.0300
	Random SNP Corruption	0.5138 ± 0.0244	
1NN imputation	Avg	0.1752 ± 0.0109	0.4624 ± 0.0255
	Rank Avg	0.2033 ± 0.0104	0.4401 ± 0.0197
	Max Per True Class Avg	0.2215 ± 0.0139	0.4613 ± 0.0202
	Max Per True Class Rank Avg	0.2377 ± 0.0158	0.4414 ± 0.0145
	Max Per Pred Class Avg	0.2317 ± 0.0179	0.4567 ± 0.0210
	Max Per Pred Class Rank Avg	0.2540 ± 0.0144	0.4429 ± 0.0151
	Random SNP Corruption	0.3569 ± 0.0082	

S4 Supplemental Methods

S4.1 SNP processing in the CaG Omni and Axiom subsets

For CaG datasets genotyped using the Axiom and Omni arrays, genotype data were first lifted over to GRCh38 using the liftOver tool from the UCSC Genome Browser [7] with the hg19ToHg38 chain file. SNPs with more than 50% missingness were removed prior to harmonization. Alleles were then aligned to the GRCh38 reference genome using bcftools fixref and Will Rayner's strand alignment tool. SNPs exhibiting allele frequency differences greater than 20% compared to TOPMed were excluded. After harmonization, these datasets were intersected with the harmonized SNP set shared between 1KGP and the CaG GSA subset to obtain the final sets.

S4.2 Self-Reported ethnicity labels in biobank cohorts

Differences in how population or ethnicity information is collected across biobanks require cohort-specific strategies for assigning population labels. In particular, biobank datasets rely on self-reported information captured through questionnaires, whose structure, terminology, and level of detail vary between studies and may not fully align with genetically defined ancestry groups. In the following sections, we describe the labeling strategies implemented for each of the CaG, MHI and AoU biobanks, based on the available metadata. It is important to note that the terminology and classification schemes used in these questionnaires were defined by the original biobank protocols and reflect prevailing standards at the time of data collection.

S4.2.1 CaG

Participants in the CaG cohort completed a self-administered socio-demographic and lifestyle questionnaire, which included questions on ethnicity and familial origin. Ethnicity was self-reported by selecting one or more of the following predefined categories: White European, Black, Arab, Latino, Southeast Asian, East Asian, West Asian, South Asian, Jewish, or Other. In addition, individuals were asked to provide information on their country of birth, as well as the countries of birth of their parents and grandparents.

Population labels were derived from the reported birth countries of participants' grandparents. These populations included individuals of Italian ancestry, closely aligned with the *Toscani in Italy* (TSI) population from 1KGP, and individuals of Chinese ancestry, who may correspond to several 1KGP reference groups, including *Han Chinese in Beijing* (CHB), *Han Chinese South* (CHS), and *Chinese Dai in Xishuangbanna* (CDX). We also evaluated model performance on individuals with African-European admixed ancestry, focusing on Haitian individuals, a population with known admixture but not represented in 1KGP. Nevertheless, similar admixed populations such as *African Caribbeans in Barbados* (ACB) and *African Ancestry in the Southwest US* (ASW) are included in 1KGP. Furthermore, we assessed predictions for French Canadians (FC), a population of European descent that constitutes the majority of the biobank but is absent from the 1KGP reference. This offered a valuable test case for evaluating model generalization to a well-sampled yet unrepresented popula-

tion. Lastly, we included a population entirely absent from 1KGP, the Moroccan population, representing North-African ancestry.

S4.2.2 MHIB

Participants in the MHIB cohort self-reported their ancestry by selecting from predefined categories described in the biobank questionnaire with the following descriptors: Caucasian (defined as having ancestors from Europe, North Africa, or the Middle East) hereafter referred to as White, Hispanic (e.g., Mexican, Puerto Rican, Cuban, South or Central American), Black (ancestors from African or Black ethnicity), Asian (ancestors from the Orient, Asia, Indian subcontinent, or Pacific Islands—including China, Japan, Philippines, Korea, and Samoa), Native American (ancestors from any North American Indigenous group or tribe), and Other. These labels and definitions reflect the original wording used in the MHIB questionnaire and associated metadata. We recognize that some of these terms may be considered outdated or sensitive due to their cultural and historical connotations. Their inclusion here is intended solely to preserve the integrity of the source data, and does not imply endorsement of these classifications.

As in CaG, individuals of Italian ancestry in MHIB were identified based on grandparental birth country information. However, due to less consistent documentation of grandparental information in MHIB, we relied on the self-reported ethnicity field to define labels for other population groups. For our analysis, we retained individuals of Asian ancestry, a broad category that can relate to both East Asian and South Asian populations in 1KGP, African ancestry, and French Canadian descent.

- 1 individual who self-identified as French Canadian (FC) but had an EUR ancestry proportion < 0.7;
- 9 individuals who self-identified as African but had an AFR ancestry proportion < 0.7;
- 4 individuals who self-identified as Asian but had an EAS or SAS ancestry proportion < 0.7.

S4.2.3 AoU

Participants in the AoU cohort self-reported their ancestry information using separate race and ethnicity fields. The race field category in the questionnaire included the descriptors Asian, Black or African American, Middle Eastern or North Africa, Native Hawaiian or Other Pacific Islander, White or More than one population. The ethnicity field category in the questionnaire, distinguished between the descriptors Hispanic or Latino and not Hispanic or Latino. For our analysis, we retained individuals self-reporting White, Black or African American, or Asian in the race field who also reported not Hispanic or Latino in the ethnicity field. Hispanic or Latino individuals were retained if they self-reported Hispanic or Latino in the ethnicity field and did not provide a race designation.

S4.3 Details of Diet Network training and cross-dataset validation

S4.3.1 Training on 1KGP data

15 Diet Network models were trained on the 1KGP dataset using 5-fold cross-validation, each fold repeated across three different random seeds to assess variability due to random initialization. The input dataset consisted of 2,322 individuals across 24 population labels. Each fold was split into training (60%), validation (20%), and test (20%) sets, ensuring that every individual appeared once in the test sets across folds. Population labels were balanced between training, validation and test splits.

For the auxiliary network, SNP embeddings were derived from genotype frequencies computed from the training set within each fold (see Figure S1). Genotype inputs to the main network were standardized by subtracting the mean and dividing by the standard deviation of each SNP, calculated across the training set.

The number of layers, neurons per layer, optimizer and loss function were kept consistent with the original Diet Network implementation [8]. A hyperparameter search was conducted to optimize the learning rates for the main and auxiliary networks using performance on the validation set.

We trained multiple models using a range of input dropout rates (0, 0.5, 0.9, 0.99, 0.9925, 0.995, 0.997, 0.999, 0.9995, 0.9999), and identified the optimal input dropout rate by evaluating classification accuracy on test sets with varying proportions of randomly masked SNPs (0.1, 0.25, 0.5, 0.7, 0.8, 0.9, 0.95, and 0.99). SNP masking was performed as follows: for missingness proportions up to 0.5, we generated non-overlapping SNP subsets to ensure each SNP was removed at least once (e.g., two non-overlapping subsets for a missingness proportion of 0.5, 100 non-overlapping subsets for a proportion of 0.01). To ensure consistent comparisons, we generated 100 subsets for each missingness proportion. For missingness proportion above 50%, subsets were instead constructed to ensure that each SNP was included at least once. Genotypes of the remaining SNPs were rescaled to account for reduced input dimensionality using a scaling factor based on the ratio of SNPs in the training and test sets (Supplemental Methods **S4.4**).

S4.3.2 Testing on 1KGP data

The 1KGP dataset was split to ensure each individual appeared in the test set exactly once across the 5 cross-validation folds. The overall performance was evaluated using predictions from the five models specific to each fold. To assess the impact of model stochasticity, the 5-folds cross-validation experiment was repeated across three different random seeds, resulting in a total of 15 models. Consequently, each individual was evaluated by three models, enabling analysis of variability due to random initialization. Model predictions are presented in Figure S3.

S4.3.3 Cross-dataset validation on CaG, MHIB and AoU

To evaluate generalizability, each individual from the CaG, MHIB and AoU datasets was tested using the 15 Diet Network models trained on 1KGP data with 99.5% input dropout. This produced 15 population label predictions per individual.

For model evaluation on the CAG GSA subset and MHIB cohort using complete SNP data, although all SNPs used during training were present, some genotypes were missing at the individual level (≤10% per SNP after SNP filtering). These missing genotypes were imputed with the mean SNP value from the corresponding 1KGP training set for each fold, which corresponds to a value of 0 after input feature standardization.

For the AoU dataset and CAG Axiom and Omni subsets, where SNP overlap with the training data was incomplete, missing values were also set to 0 following standardization, but genotypes for the remaining overlapping SNPs were rescaled to account for the reduced input dimensionality, using a scaling factor based on the ratio of SNPs in the training and test sets (Supplemental Methods S4.4).

To further assess the impact of missing SNPs on model generalizability, we performed simulations by randomly removing SNPs at different proportions of missingness (0.25, 0.50, 0.75, 0.90, 0.95, and 0.99) from the CaG GSA subset and MHIB dataset. For each proportion, one random subset of SNPs was removed, and the resulting predictions were compared to those obtained on the complete SNP data.

S4.4 Scaling missing and corrupted inputs

S4.4.1 Preliminaries and notation

Let $X = (x_1, ..., x_D)$ be a vector of size D, where each element $x_i \in (0, 1, 2)$ represents the genotype at a single SNP i. Prior to being fed into the network, each genotype x_i in X is standardized using the mean μ_i and standard deviation σ_i of the corresponding SNP, calculated from the training data. Let $Y = (y_1, ..., y_D)$ denote the vector of standardized genotypes where $y_i = \frac{x_i - \mu_i}{\sigma_i}$.
Let C denote the set of corrupted positions, let $Y' = (y'_1, ..., y'_D)$, denote the vector of data where $y'_i = y_i$ when $i \notin C$ and y'_i is corrupted when $i \in C$. We want to scale up Y' in order to keep $\sum y'_i$ as close as possible as $\sum y_i$ Assuming that:

$$\sum y_i \approx \frac{D}{|C|} \sum_{i \in C} y_i \approx \frac{D}{D - |C|} \sum_{i \notin C} y_i \tag{1}$$

S4.4.2 Scaling for missing inputs and Genotype-average corruption method

We define missing data or data corrupted with the Genotype-average corruption method Y' such that if position i is missing or corrupted, then y_i is replaced by 0. We define Y' as:

$$y'_i = \frac{D}{D - |C|} \left\{ \begin{array}{ll} y_i, & i \notin C \\ 0, & i \in C \end{array} \right\} \tag{2}$$

By scaling our data by $\frac{D}{D-|C|}$ we can then make $\sum y'_i$ closer to $\sum y_i$.

$$\sum y'_i = \frac{D}{D - |C|} \sum_{i \notin C} y_i \approx \sum y_i \tag{3}$$

S4.4.3 Scaling for reference corruption method

If our corruption involves replacing the value with the reference, then corrupting position i results in y_i being replaced by $-\frac{\mu_i}{\sigma_i}$.

A similar computation yields the scaling and translating needed. If we let μ and σ be the average mean and standard deviation of the μ_i and σ_i respectively, we define Y' as:

$$y_i' = \frac{D}{D-|C|}\left\{\begin{array}{ll} y_i & i \notin C \\ -\mu_i/\sigma_i & i \in C \end{array}\right\} + \frac{D}{|C|}\frac{\mu}{\sigma} \tag{4}$$

By scaling our data by $\frac{D}{D-|C|}$ and translating it to $\frac{D}{|C|}\frac{\mu}{\sigma}$ we can then make $\sum y_i'$ closer to $\sum y_i$.

$$\sum y_i' = \frac{D}{D-|C|}\sum_{i \notin C} y_i + \frac{D}{|C|}\frac{\mu}{\sigma} \approx \sum y_i \tag{5}$$

S4.5 Aggregating local attribution scores into global attribution scores

We use four feature attribution methods in this study, that provide local attribution scores per SNP and per individual: Saliency Maps, Integrated Gradients, DeepLIFT, and GradientSHAP. Briefly, Saliency Maps compute the gradient of the model output with respect to the input features [9]. Integrated Gradients estimate feature importance by integrating the gradients of interpolated values along a linear path between the input and one or more baselines. DeepLIFT calculates the difference in neuron activations between the input and a reference baseline, backpropagating these contribution scores through the network. GradientSHAP estimate feature importance by averaging gradients along randomly sampled paths between the input and a baseline, estimating Shapley value based on these integrated gradients.

We consolidated the local attribution scores returned by the attribution methods into a single global attribution score per SNP using several aggregation strategies, as there is currently no established consensus in the machine learning literature for converting local attributions to global importance. Specifically, we compared using average and rank average of local attribution values, with the latter aiming to reduce the influence of outliers. Additionally, we explored averaging local attribution scores across individuals grouped by either their true population or predicted population, in order to capture population-specific signal, acknowledging that certain SNPs may only be highly attributed within specific populations.

We performed a series of SNP corruption experiments in which we corrupted increasing number of SNPs based on their ranking derived from global attribution scores. In this setting, the same set of SNPs was removed for all individuals, in contrast to corruption experiments based on local attribution rankings, where the set of SNPs removed varied between individuals.

The area A_{ord} and A_{rev} metrics revealed that averaging local attributions yielded better performance than the other aggregation strategies tested (Figure S8 and Table S3). Specifically, SNP corruption based on global attribution rankings yielded lower A_{rev} values compared to random SNP corruption under the "replace with missing" corruption method. This result suggest that, in this case, less informative SNPs were more effectively identified

through random selection than by feature attribution. However, this pattern was not observed with the resample value and 1NN substitution corruption methods. We concluded that the observed worst performance of corrupting low attribution SNPs compared to random under the "replace with missing" corruption is more plausibly attributed to the stronger impact of missing values on model predictions.

References

[1] Weir, B. S. and Cockerham, C. C. Estimating f-statistics for the analysis of population structure. *Evolution*, 38(6):1358–1370, 1984. ISSN 00143820, 15585646. URL http://www.jstor.org/stable/2408641.

[2] Holsinger, K. E. and Weir, B. S. Genetics in geographically structured populations: defining, estimating and interpreting fst. *Nature Reviews Genetics*, 10(9):639–650, September 2009. ISSN 1471-0064. doi: 10.1038/nrg2611. URL http://dx.doi.org/10.1038/nrg2611.

[3] Gaspar, H. A. and Breen, G. Probabilistic ancestry maps: a method to assess and visualize population substructures in genetics. *BMC Bioinformatics*, 20(1), March 2019. ISSN 1471-2105. doi: 10.1186/s12859-019-2680-1. URL http://dx.doi.org/10.1186/s12859-019-2680-1.

[4] Sengupta, D., Choudhury, A., Basu, A., and Ramsay, M. Population stratification and underrepresentation of indian subcontinent genetic diversity in the 1000 genomes project dataset. *Genome Biology and Evolution*, 8(11):3460–3470, October 2016. ISSN 1759-6653. doi: 10.1093/gbe/evw244. URL http://dx.doi.org/10.1093/gbe/evw244.

[5] Schlauch, D., Fier, H., and Lange, C. Identification of genetic outliers due to substructure and cryptic relationships. *Bioinformatics*, 33(13):1972–1979, March 2017. ISSN 1367-4811. doi: 10.1093/bioinformatics/btx109. URL http://dx.doi.org/10.1093/bioinformatics/btx109.

[6] Fairley, S., Lowy-Gallego, E., Perry, E., and Flicek, P. The international genome sample resource (igsr) collection of open human genomic variation resources. *Nucleic Acids Research*, 48(D1):D941–D947, October 2019. ISSN 1362-4962. doi: 10.1093/nar/gkz836. URL http://dx.doi.org/10.1093/nar/gkz836.

[7] Karolchik, D., Hinrichs, A. S., and Kent, W. J. The ucsc genome browser. *Current Protocols in Bioinformatics*, 40(1), December 2012. ISSN 1934-340X. doi: 10.1002/0471250953.bi0104s40. URL http://dx.doi.org/10.1002/0471250953.bi0104s40.

[8] Romero, A., Carrier, P. L., Erraqabi, A., Sylvain, T., Auvolat, A., Dejoie, E., Legault, M.-A., Dubé, M.-P., Hussin, J., and Bengio, Y. Diet networks: Thin parameters for fat genomics. In *Proceedings of the International Conference on Learning Representations (ICLR)*, 2017. URL https://arxiv.org/abs/1611.09340. arXiv:1611.09340.

[9] Simonyan, K., Vedaldi, A., and Zisserman, A. Deep inside convolutional networks: Visualising image classification models and saliency maps. In Bengio, Y. and LeCun, Y., editors, *2nd International Conference on Learning Representations, (ICLR) 2014, Banff, AB, Canada, April 14-16, 2014, Workshop Track Proceedings*, 2014. URL http://arxiv.org/abs/1312.6034.
